# The CYP71AZ P450 Subfamily: A Driving Factor for the Diversification of Coumarin Biosynthesis in Apiaceous Plants

**DOI:** 10.3389/fpls.2018.00820

**Published:** 2018-06-19

**Authors:** Célia Krieger, Sandro Roselli, Sandra Kellner-Thielmann, Gianni Galati, Bernd Schneider, Jérémy Grosjean, Alexandre Olry, David Ritchie, Ulrich Matern, Frédéric Bourgaud, Alain Hehn

**Affiliations:** ^1^Laboratoire Agronomie et Environnement, Institut National de la Recherche Agronomique, Université de Lorraine, Nancy, France; ^2^Institut für Pharmazeutische Biologie und Biotechnologie, Philipps-Universität Marburg, Marburg, Germany; ^3^Max Planck Institute for Chemical Ecology, Jena, Germany; ^4^INRIA Nancy, Grand-Est Research Centre, Laboratoire Lorrain De Recherche En Informatique Et Ses Applications, Nancy, France; ^5^Plant Advanced Technologies, Nancy, France

**Keywords:** coumarin, cytochrome P450, furanocoumarin, *Pastinaca sativa*, psoralen, specialized metabolism

## Abstract

The production of coumarins and furanocoumarins (FCs) in higher plants is widely considered a model illustration of the adaptation of plants to their environment. In this report, we show that the multiplication of cytochrome P450 variants within the CYP71AZ subfamily has contributed to the diversification of these molecules. Multiple copies of genes encoding this enzyme family are found in Apiaceae, and their phylogenetic analysis suggests that they have different functions within these plants. CYP71AZ1 from *Ammi majus* and CYP71AZ3, 4, and 6 from *Pastinaca sativa* were functionally characterized. While CYP71AZ3 merely hydroxylated esculetin, the other enzymes accepted both simple coumarins and FCs. Superimposing *in silico* models of these enzymes led to the identification of different conformations of three regions in the enzyme active site. These sequences were subsequently utilized to mutate CYP71AZ4 to resemble CYP71AZ3. The swapping of these regions lead to significantly modified substrate specificity. Simultaneous mutations of all three regions shifted the specificity of CYP71AZ4 to that of CYP71AZ3, exclusively accepting esculetin. This approach may explain the evolution of this cytochrome P450 family regarding the appearance of FCs in parsnip and possibly in the Apiaceae.

## Introduction

Plants have developed a wide range of strategies to take advantage of their environment and to adapt to many conditions by methods such as the production of defense molecules. This process has been studied for many decades (Fraenkel, 1959), but the molecular characterization of the enzymes responsible for the synthesis of the defense molecule arsenal primarily started in the 1990s (Funk and Croteau, 1993; Werck-Reichhart et al., 1997; Glawischnig et al., 1999). Beside dioxygenases (Kawai et al., 2014), the cytochrome P450s (P450s) are the most diversified enzyme family in plants (Nelson et al., 2004; Nelson and Werck-Reichhart, 2011) and are estimated to represent up to 1% of the annotated genes in plant genomes (Mizutani and Ohta, 2010). Because of their diversity, these enzymes are considered major players in boosting metabolic pathway activity and therefore defense molecules (Glawischnig et al., 1999; Irmisch et al., 2014). Thanks to the development of next-generation sequencing methods, the number of transcriptomic databases has exploded. However, although the number of annotated P450 sequences is increasing day by day, the precise P450 functions often remain unknown. For example, more than 70% of the 245 P450s in the *Arabidopsis* genome, which is smaller than many other plant genomes, are orphans at the biochemical level (Mizutani and Sato, 2012).

**FIGURE 1:**
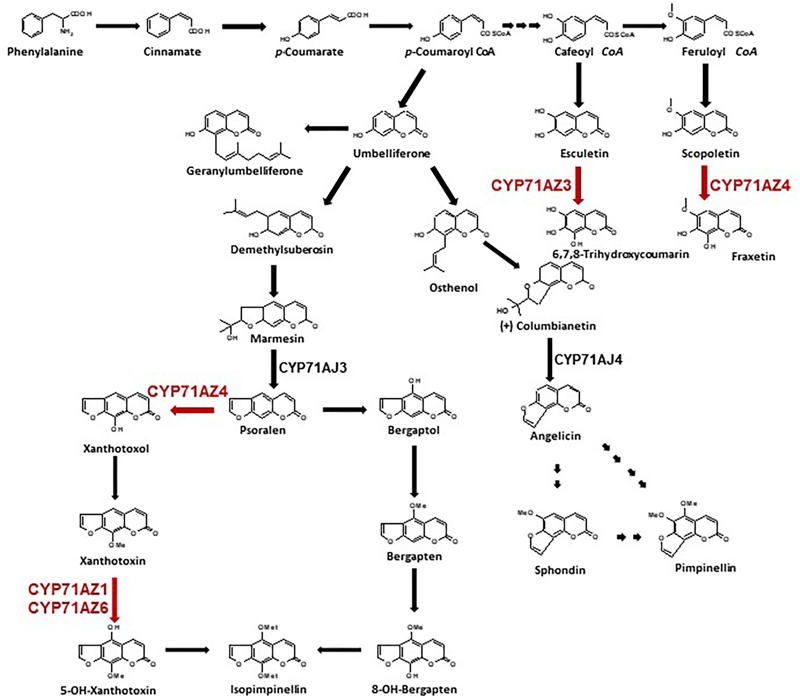
Biosynthetic pathway of furanocoumarins and coumarins. Steps in red are characterized in this study. CYP71AJ3 is the psoralen synthase (Larbat et al., 2007), CYP71AJ4 is the angelicin synthase (Larbat et al., 2009).

Furanocoumarins (FCs) are molecules involved in defense reactions against attacks by herbivores (Berenbaum et al., 1991; Li et al., 2004). They have been detected in only a few plant families, and these families are not phylogenetically related. Linear (psoralens) and angular FCs may have arisen by convergent evolution (Bourgaud et al., 2014) and are distinguished by the position of the furan-ring on the coumarin core molecule (**Figure 1**). Plants first acquired the ability to produce linear isomers, and the formation of the angular analogs evolved later (Berenbaum, 2002). No plant that can synthesize only angular molecules has been described. Elicitor treatment of *A. majus* cell cultures increased the synthesis of FCs and the conversion of psoralen to 5-hydroxypsoralen (bergaptol) (Hamerski and Matern, 1988a), and the use of radiolabeled precursors assigned the enzymatic conversion of demethyl [3-^14^C]suberosin into labeled (+) marmesin (Hamerski and Matern, 1988b) to P450s. However, only a few of these enzymes have been isolated and identified. The first P450 entity that was characterized at the molecular level catalyzes the synthesis of psoralen from (+)-marmesin and was assigned to the CYP71AJ P450 subfamily (Larbat et al., 2007). A variant in parsnip named CYP71AJ4 and sharing 70% amino acid identity with psoralen synthases did not accept marmesin as a substrate and was the first P450 shown to be involved in angular FC biosynthesis, converting (+)-columbianetin to angelicin (Larbat et al., 2009) (**Figure 1**). The assignment of two different P450s to the same subfamily but restricted to only one biosynthetic pathway each suggested that these enzymes evolved from a common ancestor (Larbat et al., 2009). A CYP71AJ-targeted search in various Apiaceae transcriptomic databases identified 36 partial or full-length paralogous genes distributed in 11 different apiaceous plants that either produce or lack FCs (Dueholm et al., 2015). A phylogenetic analysis of 19 of these CYP71AJ sequences revealed a clustering of 5 enzymes that have been described to be involved in the synthesis of FCs (Larbat et al., 2007, 2009; Dueholm et al., 2015). The functions of the 14 remaining enzymes have not been elucidated. This analysis suggests that the CYP71AJ P450 family might have evolved to synthesize these toxic defense molecules. An ancestral version of these enzymes might have been identified in plants that do not produce FCs (Dueholm et al., 2015). The spread of this gene family probably occurred through gene duplication associated with neofunctionalization. Such a mechanism has been described, for example, in *Arabidopsis* and for the glucosinolate biosynthesis pathway (Kliebenstein et al., 2001; Benderoth et al., 2006; Kliebenstein and Osbourn, 2012; Edger et al., 2015).

Here, we describe the identification and the functional characterization of several members of the CYP71AZ P450 subfamily. *CYP71AZ1* was isolated by using a differential display RT-PCR approach applied to *A. majus in vitro* cultured cells that were treated with *Phytophthora sojae* extracts. CYP71AZ1 shares 33.8% identity with the psoralen synthase CYP71AJ3. BLAST searches performed on transcriptomic and genomic databases led to the identification of numerous paralogous genes that interestingly (and except one) were restricted to Apiaceae. In this report, we focused on four genes that were identified in parsnip and share 63 to 85% identity with each other. An *in vivo* investigation revealed that these four genes have different expression patterns in wounded parsnip roots. An *in vitro* functional characterization using proteins expressed in yeast showed that these enzymes are involved in the synthesis of both FCs and coumarins. A molecular shuffling of regions that change the catalytic sites of CYP71AZ4 allowed us to transform a FC/coumarin non-specific enzyme to a coumarin-specific enzyme. This study provides evidence that this multigenic P450 subfamily contributed to the diversification of coumarins and provides new insight into the evolution of higher plants.

## Materials and Methods

### *Ammi majus* Culture and Isolation of CYP71AZ1

Total RNA and differential amplification of 3′-fragments of P450 cDNAs from elicited or water-treated (control) *A. majus* cell cultures was done as already described previously (Larbat et al., 2007).

### Parsnip Culture

Parsnips (*Pastinaca sativa* L. subsp. sativa “Demi Long de Guernesey”) were cultivated for 2 months as described by Munakata et al. (2016). Leaves and roots were separated from six plants used as controls and another six that endured the mechanical elicitation treatment as described by Roselli et al. (2016). Seeds were germinated in a growth chamber in soil under regulated climatic conditions, namely, temperature: 20/24°C; hygrometry: 40/70%; and light: 16 h/8 h day/night. Then, germinated seeds were transplanted into soil pots at the two leaf stage for an additional 2 months growth with the same climatic conditions.

### Wounding Induction

Plants were removed from the soil, and the roots were carefully washed with distilled water and dried. Wounding was done uniformly with a metal tip on the surfaces of the leaves, roots, and stems. Wounded tissues were frozen in liquid nitrogen and crushed with a mortar and a pestle prior to polyphenol extraction.

### Real-Time Quantitative PCR of the Target Gene Transcripts

Total RNA extraction from plants cultivated in soil was performed according the E.Z.N.A. Plant RNA kit protocol (Omega Bio-Tek, Norcross, GA, United States). Remaining DNA was digested using an Amplification Grade DNAse kit (Sigma-Aldrich, Saint-Louis, MO, United States^1^). The cDNA was synthetized using a High-Capacity cDNA Reverse Transcription Kit (Thermo Fisher Scientific, Waltham, MA, United States^2^) and 100 ng of extracted mRNA. The cDNA was diluted fivefold before real-time quantification (StepOnePlus, Applied Biosystems). The real-time quantification was performed using a SYBR Green Master Mix: SYBR *Premix Ex Taq II* (Tli RNase H Plus) kit (Clontech, Mountain View, CA, United States^3^). The experiments were done on six biological replicates each.

The primers used for real-time quantification were as follows: 5.8S for 5′-AGCGGCGTCTTTCCAAAAC-3′ and 5.8S rev 5′-ATATCTCGGCTCTCGCATCG-3′ for the house-keeping gene 5.8S; CYP71AZ3for 5′-TGACGACCGTAGAGCTTGCA-3′ and CYP71AZ3rev 5′-ATTGGGAACCACCTGCTGG-3′ for CYP71AZ3; CYP71AZ4for 5′-GGAAAAGAGAAATTCCAACGAATG-3′ and CYP71AZ4rev 5′-TGTTTGCTGCCTTTTTTGTTTC-3′ for CYP71AZ4; CYP71AZ6for 5′-GTTTGGGCGATGACAGCA-3′ and CYP71AZ6rev 5′-TGTCTTCCTGGGCTTTCTTCA-3′ for CYP71AZ6; and CYP71AJ3for 5′-AACAATAGCGACGGCTTTGG-3′ and CYP71AJ3rev 5′-TTAAAAGTCCGGACGCCATG-3′ for CYP71AJ3.

### Cloning of the CYP71AZ1 Open Reading Frame

The CYP71AZ1 open reading frame was PCR-amplified using a high-fidelity DNA polymerase (PrimeSTAR Max; Clontech) and primers encoding an additional 6xHis tag at the 3′ end (CYP71AZ1for 5′-ATGCAGATGGATGCAGTAGTTATCCTTCTTATTC-3′ and CYP71AZ1rev 5′-TTA*GTGGTGATGGTGATGATG*TGAAAACATATATG-3′). PCR conditions were as follows: 5 min at 98°C, 30 cycles (10 s at 98°C, 10 s at 55°C, and 120 s at 72°C), and a final 5 min extension step at 72°C. PCR products were purified, cloned using a pCR^®^8/GW/TOPO^®^ TA Cloning^®^ Kit (Invitrogen^®^), and sequenced. The resulting PCR product cloned in pCR^®^8/GW/TOPO^®^ was sequenced and then subcloned in the yeast expression vector pYeDP60-GW (Dueholm et al., 2015) using LR clonase II (Invitrogen).

### Cloning of CYP71AZ3, CYP71AZ4, and CYP71AZ6 Open Reading Frames

Total RNA was extracted from the leaves of parsnip 6 h after mechanical wounding (Larbat et al., 2009; Roselli et al., 2016) with the RNeasy plant extraction kit (Qiagen). The cDNA was generated using a High-Capacity RNA-to-cDNA system (Applied Biosystems) and random primers. The full-length cDNA sequence was then PCR-amplified using Platinum^®^ *Taq* DNA Polymerase High Fidelity (Invitrogen) and primers with additional restriction sites and a 6xHis tag sequence at the 3′ end: CYP71AZ3for 5′-GGATCCATGGAGCCAGTATTTCTCTTTC-3′, CYP71AZ3rev 5′-GAATTCTTA*GTGGTGATGGTGATGATG*TGGAAACATATATTTTTTAGG-3′, CYP71AZ4for 5′-GGTACCATGGATCCAGCAGCTATC-3′, CYP71AZ4rev 5′-GAATTCTTA*GTGGTGATGGTGATGATGTG*GATGTACATATATTTTAGG-3′, CYP71AZ6 for 5′-ATGGATCCAGTAGTTATCTTTCTTGTCCTTGCTT-3′, and CYP71AZ6rev 5′-TTATGGACATATATATTTTTTAGGTCGAATGTAG-3′. PCR conditions were as follows: 2 min at 94°C, 35 cycles (30 s at 94°C, 30 s at 55°C, and 2 min at 68°C), and a final 10 min extension step at 68°C. PCR products were purified, cloned using a pCR^®^8/GW/TOPO^®^ TA Cloning^®^ Kit (Invitrogen), and sequenced. The resulting PCR products cloned in pCR^®^8/GW/TOPO^®^ were sequenced and finally subcloned in the yeast expression vector pYeDP60-GW (Dueholm et al., 2015) using LR clonase II (Invitrogen).

### Expression in a Yeast System

Recombinant plasmids and an empty plasmid (negative control) were used to transform the *S. cerevisiae* strain WAT11, which expresses the *Arabidopsis thaliana* NADPH P450 reductase 1 (Pompon et al., 1996), except for pYeDP60-GW::*CYP71AZ1*, which was used to transform the *S. cerevisiae* strain WAT21, which expresses the *A. thaliana* NADPH P450 reductase 2. Microsome preparation and enzyme assays were conducted as described previously (Larbat et al., 2007). The molecules tested are listed in **Supplementary Figure S1**. The catalytic parameters are the means of three independent replicates, and errors represent standard deviations. The kinetic parameters were calculated using the SigmaPlot 12 program (Systat Software Inc.).

### Metabolite Analysis

The roots of 2-month-old *P. sativa* var. Demi Long de Guernesey were harvested, frozen in liquid nitrogen and ground in a mortar with a pestle. The protocol to prepare the samples and analyze the FC content by ultrahigh performance liquid chromatography (UHPLC)/MS was derived from Munakata et al. (2016).

### LTQ Orbitrap

The analytical column was a Grace-Discovery Alltima C18 (150 mm × 4.6 mm, 5 μm) reversed-phase column. The mobile phases used for the separation consisted of distilled water with 0.1% formic acid (A) and methanol with 0.1% formic acid (B). The samples were analyzed in the following gradient mode: from 0 to 1 min, 10%B, increasing B concentration to 70% at 20 min and 100% at 22 min. The mobile phase was held constant at 100%B between 22 and 25 min and then back at beginning conditions during 45 min. All samples were injected with a volume of 25 μL, and the mobile phase flow rate was kept at 0.2 mL/min. The HPLC/MS system was composed of a quaternary solvent delivery pump and linear ion trap mass spectrometer (LTQ-MS, Thermo Electron Corporation, Waltham, MA, United States) coupled with an Orbitrap HRMS (Thermo Electron Corporation, Waltham, MA, United States). The LTQ-Orbitrap was equipped with an electrospray ionization (ESI) interface operating in positive ion mode. The orbitrap was used in scan mode between 80 and 400 m/z, and the 10 most intense selected masses were analyzed in MS/MS using LTQ fragmentation.

### Ultrahigh Performance Liquid Chromatography-MS

The analytical column was a Phenomenex Kinetex XB-C18 (150 mm × 2.1 mm, 2.6 μm) reversed-phase column. The mobile phases used for the separation consisted of distilled water with 0.1% formic acid (A) and methanol with 0.1% formic acid (B). The samples were analyzed in gradient mode as follows (A/B; v/v): 90:10 at 0 min, 80:20 at 0.74 min, 40:60 at 5.88 min, 10:90 at 10 min, 0:100 between 12 and 16 min, and 90:10 from 16.01 to 20 min. All samples were injected with a volume of 3 μL, and the mobile phase flow rate was kept at 0.2 mL/min. The column was kept at 40°C during the runtime. The UHPLC/MS system was composed of a Shimadzu Nexera UHPLC (pump, automatic sampler, and PDA analyzer, Shimadzu Corp, Kyoto, Japan) coupled with a Shimadzu LCMS2020 single quadrupole mass detector (Shimadzu Corp., Kyoto, Japan). MS was used with an ESI interface operating in positive ion mode.

### Nuclear Magnetic Resonance Spectroscopy

^1^H NMR, ^1^H-^1^H COSY, and HSQC spectra were recorded on an Avance III HD 700 NMR spectrometer (Bruker-Biospin, Karlsruhe, Germany) equipped with a 1.7 mm TCI microcryoprobe. Spectra were measured at 300 K and referenced to the residual solvent signals of MeOH-*δ_4_* (δ_H_ 3.31 and δ_C_ 49.15) and acetone-*δ_6_* (δ_H_ 2.05 and δ_C_ 29.92). Coupling constants are in Hertz (Hz). Data acquisition and processing were accomplished using TopSpin 3.2. Standard pulse programs as implemented in TopSpin were used for data acquisition.

### Modeling

A library of 1,023 three-dimensional (3D) structures of CYP450 domains (Pfam code PF00067) was extracted from the KBDOCK database (Ghoorah et al., 2014), which had been built from a June 2013 snapshot of the Protein Databank (PDB). A sequence-based search of these structures using version 3.0 of the Kpax program (Ritchie et al., 2012) yielded the PDB structure 4EJI (P450 2A13) as the closest sequence homolog to CYP71AZ (39% sequence identity). Four similar structures (PDB codes 3QU8, 3T3Z, 3TK3, and 2NNJ) having no more than 90% sequence similarity to 4EJI were then extracted from the library using a structure-based search in Kpax. A multiple structure alignment of the first selected structures was built with Kpax, and the resulting multiple sequence alignment profile was used as input to the MODELLER (v9.13) homology modeling program (Sali and Blundell, 1993). Ten structural models were built using default MODELLER parameters, and the structure with the best DOPE score was retained as the final model of the CYP71AZ structure.

### *In Silico* Data Mining

The presence of various CYP71AZs was investigated, and their sequences were collected in transcriptomic and genomic databases [*Angelica archangelica* (onekp), *Apium graveolens* (SRR1023730 and DRR003696), *Bupleurum chinense* (SRR1002958), *Bupleurum scorzonerifolium* (SRR1002959), *Centella asiatica* (Phytometasyn), *Cimicifuga racemosa* (onekp), *Coriandrum sativum* (SRR1700630, SRR1700819, and SRR1700873), *Daucus carota* (SRR187755-1^4^), *Oenanthe javanica* (SRR1119291), *Petroselinum crispum* (SRR1030614), *P. sativa* (Dueholm et al., 2015; Roselli et al., 2016), *Thapsia garganica* (Henrik Toft Simonsen), and *Conium maculatum* (Heiko Rischer and (Hotti et al., 2015)]. Sequences are available in **Supplementary Figure S10**. The single reads of Illumina and 454 databases were assembled using the CLC genomic workbench (Qiagen).

The protein sequences were compared using Clustal X, and phylogenetic trees were constructed using the maximum likelihood (ML) methods included in MEGA7.

## Results

### Identification of a Novel P450 Subfamily: CYP71AZ

Various studies have reported the pivotal role of P450 enzymes in the synthesis of FCs (Hamerski and Matern, 1988a,b; Larbat et al., 2007, 2009). To identify new genes involved in the synthesis of these molecules, we used a differential expression strategy that has been described previously (Larbat et al., 2007). A reverse transcription differential display approach using primers targeting a conserved P450 sequence identified a partial gene that was expressed more in *A. majus* cell cultures elicited with crude *Phytophthora* cell wall elicitors than non-elicited cells. The full-length sequence was cloned using rapid amplification of cDNA ends (RACE) experiments, and the deduced protein sequence was submitted to the international P450 nomenclature committee and assigned as CYP71AZ1, the first member of a novel P450 subfamily (GenBank ABO32529.1)

**FIGURE 2:**
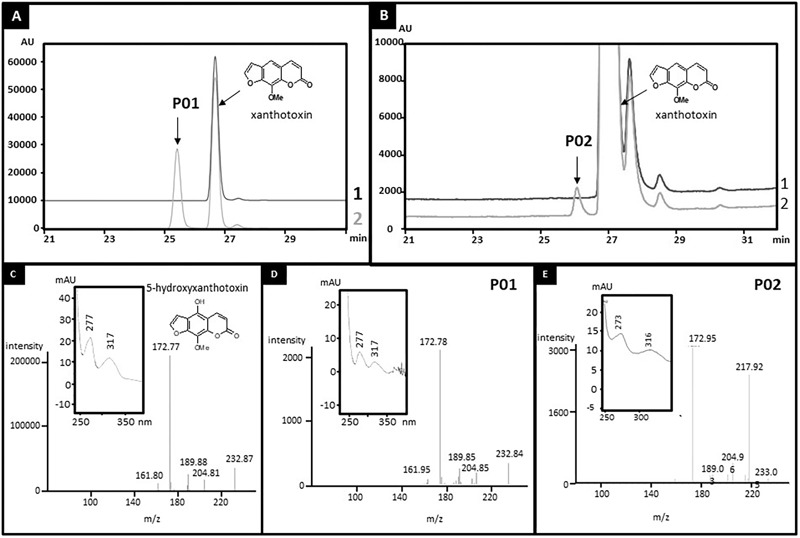
Identification of the products of xanthotoxin metabolization by CYP71AZ1 **(A,D)** and CYP71AZ6 **(B,E)**. Yeast microsomes expressing CYP71AZ1 or CYP71AZ6 convert xanthotoxin to 5-hydroxyxanthotoxin (P01 and P02). **(A)** HPLC profile of enzyme assays containing xanthotoxin, without (line 1) or with (line 2) NADPH, oxygen, and yeast microsomes expressing CYP71AZ1. **(B)** HPLC profile of enzyme assays containing xanthotoxin, without (line 1) or with (line 2) NADPH, oxygen, and yeast microsomes expressing CYP71AZ6. **(C)** MS and absorption spectrum of 5-hydroxyxanthotoxin standard. **(D)** MS and absorption spectrum of P01. **(E)** MS and absorption spectrum of P02.

CYP71AZ1 was expressed in a heterologous yeast expression system (Pompon et al., 1996) for functional characterization. A metabolic screening done with microsomes prepared from the yeast cells overexpressing this enzyme in the presence of FC pathway intermediates (**Figure 1**) showed the transformation of xanthotoxin into a single product, P01 (**Figure 2A**). Comparison with a set of standard compounds revealed that the elution time in HPLC and the UV spectrum of P01 matched those of 5-hydroxyxanthotoxin (**Figure 2C**), which is a precursor of isopimpinellin. The identity of the molecule was further confirmed by mass spectrometric analysis (**Figure 2D**). The apparent affinity for xanthotoxin (*K*_m_ = 13.1 ± 3.2 μM) is similar to the substrate affinities of most P450s involved in the synthesis of secondary metabolites. The isolation of *CYP71AZ1* paved the way to a new class of hydroxylases relevant to the metabolism of FCs.

### CYP71AZ: A Multigenic P450 Subfamily

**FIGURE 3:**
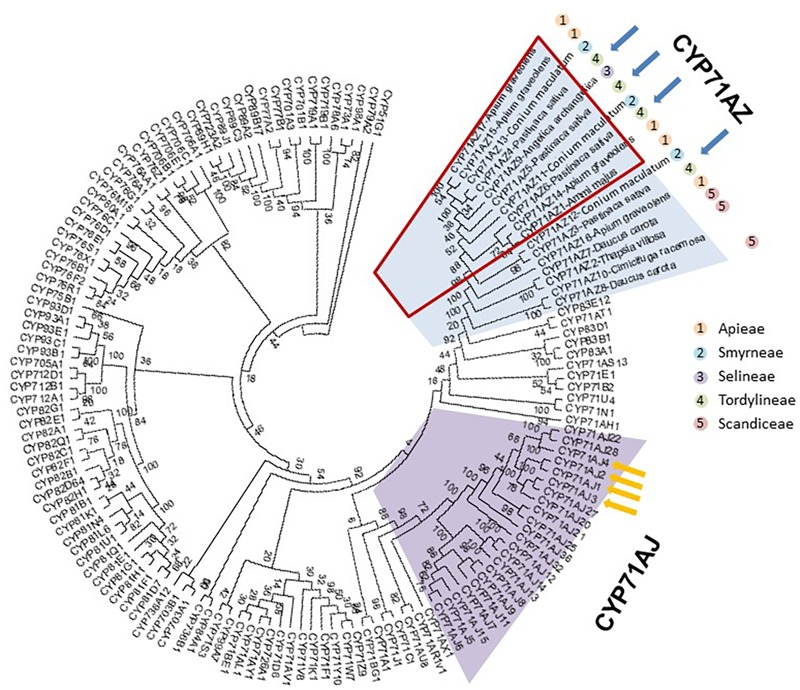
Molecular phylogenetic analysis by maximum likelihood method of the CYP71 clan. The sequences for the different P450s of clan 71 were collected in the cytochrome P450 database (http://drnelson.uthsc.edu/cytochromeP450.html and Nelson and Werck-Reichhart, 2011). The CYP71AZ sequences were collected from various databases: *A. archangelica* (onekp), *A. graveolens* (SRR1023730 and DRR003696), *B. chinense* (SRR1002958), *B. scorzonerifolium* (SRR1002959), *C. asiatica* (Phytometasyn), *C. racemosa* (onekp), *C. sativum* (SRR1700630, SRR1700819, and SRR1700873), *D. carota* (SRR187755-1 and http://apiaceae.njau.edu.cn), *O. javanica* (SRR1119291), *P. crispum* (SRR1030614), *P. sativa* (Dueholm et al., 2015; Roselli et al., 2016), *Thapsia garganica* (provided by Henrik Toft Simonsen), and *Conium maculatum* [provided by Heiko Rischer and (Hotti et al., 2015)]. Blue arrows show CYP71AZs identified in *P. sativa*. Yellow arrows show the CYP71AJ that were functionally characterized and dedicated to the synthesis of furanocoumarins. The red square shows a cluster of 10 genes; most of these genes are involved in the synthesis of furanocoumarins. The tree was rooted with CYP51G1. The evolutionary history was inferred by using the maximum likelihood method based on the JTT matrix-based model. The bootstrap consensus tree inferred from 50 replicates is taken to represent the evolutionary history of the taxa analyzed. Branches corresponding to partitions reproduced in fewer than 50% of the bootstrap replicates are collapsed. Initial tree(s) for the heuristic search were obtained automatically by applying neighbor-joining and BioNJ algorithms to a matrix of pairwise distances estimated using a JTT model and then selecting the topology with a superior log likelihood value. The analysis involved 141 amino acid sequences. All positions with less than 95% site coverage were eliminated, that is, fewer than 5% alignment gaps, missing data, and ambiguous bases were allowed at any position. A total of 388 positions were present in the final dataset. Evolutionary analyses were conducted in MEGA7.

Databases were searched for the presence of *CYP71AZ* paralogs to assess the occurrence and diversity of this P450 subfamily. Coding sequences sharing high homologies with *CYP71AZ1* are fairly restricted to a few spermatophytes and, except for *C. racemosa* (Ranunculaceae), were specifically reported in apiaceous plants such as *A. archangelica, P. sativa, D. carota, Heracleum lanatum, A. graveolens, B. chinense, B. scorzonerifolium, C. asiatica, C. sativum, O. javanica, P. crispum, C. maculatum*, and *T. garganica*. This search revealed 17 partial and full-length sequences sharing more than 55% identity and therefore belonging to the CYP71AZ subfamily (Werck-Reichhart et al., 2000). Among these 17 genes, 14 were identified in Apiaceae members known to produce FCs. However, some of them were also identified in transcriptomes of plant species that do not produce FCs, i.e., *Thapsia* spp. (Apiaceae). This result suggested that the substrate specificities of CYP71AZ-related enzymes might not be restricted to xanthotoxin (as for CYP71AZ1) and could instead provide a wider range of functions. This assumption is reinforced through a phylogenetic analysis of P450s belonging to Clan71, which shows that 10 CYP71AZ genes, including CYP71AZ1, are grouped in a cluster that were only identified in FC-producing plants (**Figure 3**). The genes identified in plants that do not produce FCs are localized out of this group. To investigate the blooming of this cluster, we further focused on *P. sativa*, which harbors four different genes. Three of them are included in the cluster and one is outside. CYP71AZ3, 4, 5, and 6 share 81.1, 71.1, 76.6, and 85.4% protein sequence identity with *A. majus* CYP71AZ1, respectively (**Supplementary Table S1**). GenBank entries for CYP71AZ3, 4, 5, and 6 are MH000218, MH000219, MH000220, and MH000221, respectively.

### *In Situ* Expression of *CYP71AZ* in Parsnip Plants Suggests Their Role in the Synthesis of FCs

**FIGURE 4:**
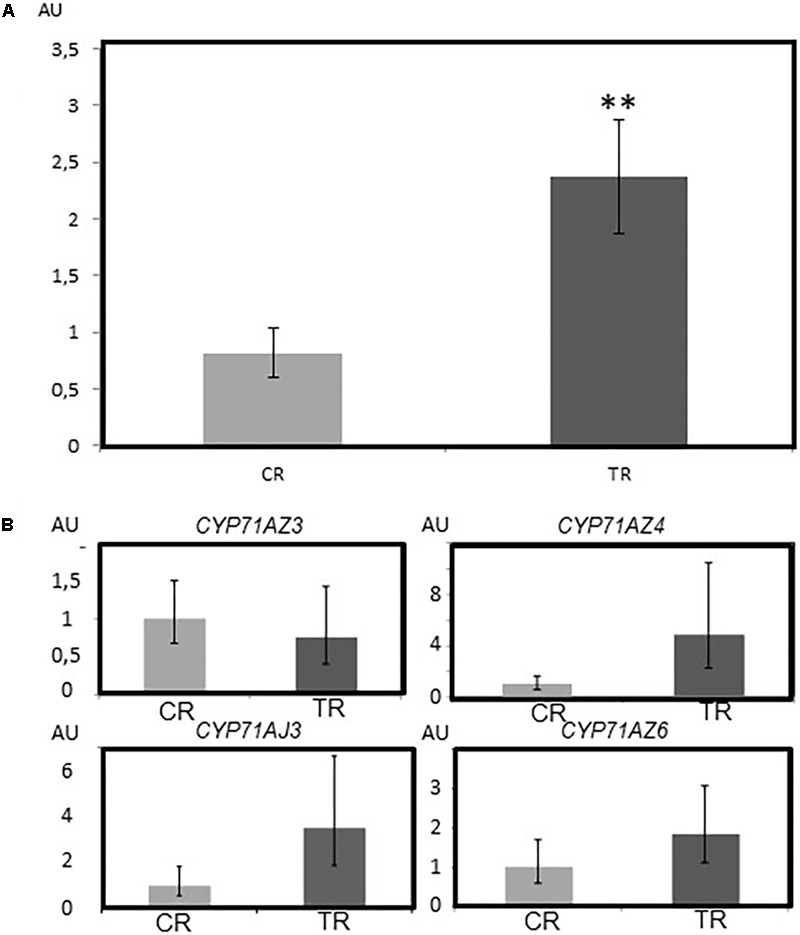
Accumulation of furanocoumarins in parsnip roots and corresponding expression pattern of *CYP71AJ3, CYP71AZ3, CYP71AZ4*, and *CYP71AZ6*. **(A)** Assessment of the FC content based on UHPLC/MS as described by Munakata et al. (2016). **(B)** mRNA expression patterns of *CYP71AZ3, CYP71AZ4, CYP71AZ6, a*nd *CYP71AJ3*. The stars represent the significant differences in the results between the two conditions according to the Wilcoxon test (^∗∗^*P*-value < 0.01). AU: arbitrary units. CR: control Roots (light gray). TR: treated roots (dark gray). The experiments were done on six biological replicates.

The metabolism of FCs in plants is influenced by environmental factors. Assessing the relationship between the concentration of FCs and the expression level of these different genes in *P. sativa* plants upon mechanical wounding was therefore relevant. In agreement with previously reported data (Roselli et al., 2016), the global FC concentration was higher in wounded roots than in unwounded roots (**Figure 4A**). The expression levels of the 4 *CYP71AZ* mRNAs were assessed and compared to the expression level of *CYP71AJ3*, which is involved in the synthesis of FCs in *P. sativa* (Larbat et al., 2007). RT-qPCR experiments (**Figure 4B**) highlighted different patterns of transcription for the four genes but these differences were not statistically relevant. The differential expression level of *CYP71AJ3* was already demonstrated by Roselli et al. (2016). The tendency of the expression profiles of *CYP71AZ4* and *CYP71AZ6* matched that of *CYP71J3*. In contrast, the expression pattern of *CYP71AZ3* was not modified in wounded plants. Although the coding sequence of *CYP71AZ5* is characterized in a genomic library (Roselli et al., 2016), we failed to amplify a full-length coding sequence corresponding to this gene. We concluded that this gene is not expressed in *P. sativa* tissues under our experimental conditions. Although not absolute proof, the diversity of the expression pattern of *CYP71AZ* genes suggests different roles in plant metabolism for the corresponding enzymes.

### Functional Characterization of CYP71AZs

**FIGURE 5:**
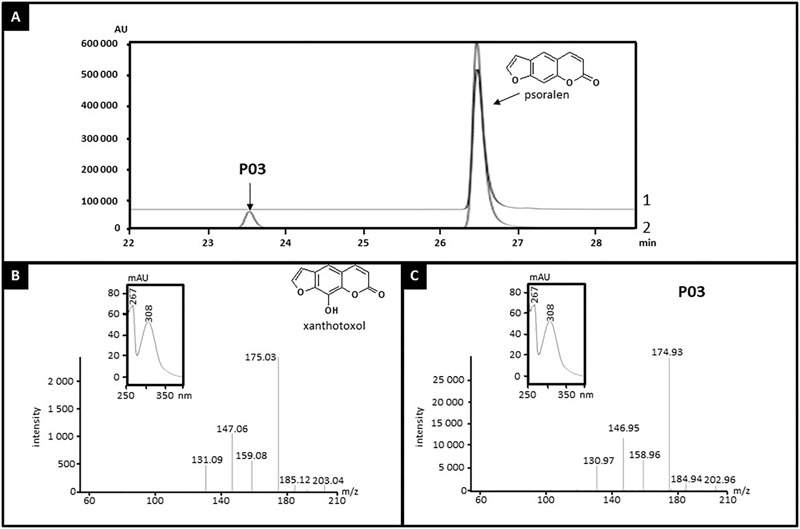
Identification of the product of psoralen metabolization by CYP71AZ4. Yeast microsomes expressing CYP71AZ4 convert psoralen to xanthotoxol (P03). **(A)** HPLC profile of enzyme assays containing psoralen, without (line 1) or with (line 2) NADPH, oxygen, and yeast microsomes expressing CYP71AZ4. **(B)** MS and absorption spectrum of standard xanthotoxol. **(C)** MS and absorption spectrum of P03.

CYP71AZ3, CYP71AZ4, and CYP71AZ6 were expressed in yeast cells, and their enzymatic activities were assayed using different intermediates of the FC pathway as substrates (**Figure 1**). CYP71AZ6 displayed the same enzymatic activity as CYP71AZ1, transforming xanthotoxin to 5-hydroxyxanthotoxin (product P02) with an apparent *K*_m_ of 6.3 ± 1.2 μM (**Figures 2B,E** and **Table 1**). This result was expected since CYP71AZ1 and CYP71AZ6 are very close from a phylogenetic point of view (**Figure 3**). As for CYP71AZ4, the enzymatic data provided evidence that this prefers psoralen as a substrate (**Figure 5A**), with a substrate affinity (*K*_m_) of 11.3 ± 2.2 μM (**Table 1**), and mass spectrometric evidence confirmed hydroxylation at the C8 position of the coumarin core structure occurred from xanthotoxol (P03; **Figures 5B,C**). Taken together, these first elements amend our knowledge of the psoralen metabolism: CYP71AZ4 catalyzes the step preceding *O*-methylation, and CYP71AZ1 and 6 perform the subsequent hydroxylation en route to isopimpinellin (**Figure 1**).

**Table 1:** Metabolization pattern and kinetic parameters for CYP71AZs.

Substrate	Product	Apparent affinity (μM)			
		CYP71AZ1	CYP71AZ3	CYP71AZ4	CYP71AZ6
Xanthotoxin	5-hydroxyxanthotoxin	P01: 13.1 ± 3.2	N.D.	N.D.	P02: 6.3 ± 1.2
Psoralen	Xanthotoxol	N.D.	N.D.	P03: 11.3 ± 2.2	N.D.
6-Methoxycoumarin	Scopoletin	P11: N.Q.	N.D.	P04: 9.5 ± 1.5	P12: N.Q.
	*m/z* = +16	N.D.	N.D.	P05: 44.8 ± 16.3	N.D.
Scopoletin	Fraxetin	N.D.	N.D.	P06: 762.2 ± 586.4	N.D.
7-Methoxy-3-methylcoumarin	*m/z* = +16	N.D.	N.D.	P07: 159.9 ± 41.1	N.D.
7-Methoxycoumarin	Daphnetin-7-methylether	P09: N.Q.	N.D.	P08: 72.5 ± 15.2	P10: N.Q.
Esculetin	*m/z* = +16	N.D.	P13: 248.6 ± 51.9	N.D.	N.D.

*Reactions were set up in KP_*i*_ buffer (pH 7.4) at 28°C for 10 min and stopped by the addition of 10 μL of 0.1 M HCl. Products were quantified after HPLC separation at 330 nm. N.D., not detected; N.Q., not quantifiable.*

In this first set of assays with FC substrates, we could not ascribe any enzyme activity to CYP71AZ3. The metabolic screening for substrates was therefore extended to simple coumarins (**Supplementary Figure S1**). Some coumarins are precursors and represent the core of FCs. This additional screening was done on all the CYP71AZs. The extended screening revealed that at least five coumarins beside psoralen served as substrates for CYP71AZ4 (**Supplementary Figures S2–S4**), although the kinetic constants determined *in vitro* for some of these compounds were higher than commonly expected for P450s and appear to preclude an *in situ* role as substrates. 6-Methoxycoumarin was converted to two products (**Supplementary Figure S2A**), one of which (P04) was identified as scopoletin (**Supplementary Figures S2B,C**, *K*_m_ = 9.5 ± 1.5 μM, **Table 1**), while the other (P05) remained unassigned but showed a maximal UV absorption shift at shorter wave length than its precursor (**Supplementary Figure S2D**, *K*_m_ = 44.8 ± 16.3 μM, **Table 1**). The CYP71AZ4-encoded hydroxylase slowly converted scopoletin to a product (P06) identified as fraxetin (**Supplementary Figures S3A–C**, *K*_m_ = 762.2 ± 586.4 μM, **Table 1**). Moreover, 3-methyl-7-methoxycoumarin and 7-methoxycoumarin also served as substrates for CYP71AZ4, yielding product P07 (**Supplementary Figure S4A**) and P08 (**Supplementary Figure S4B**), respectively, which were recognized as 8-hydroxy-3-methyl-7-methoxycoumarin (**Supplementary Figures S4C, S5**) and daphnetin-7-methyl ether (**Supplementary Figures S4D,E**), respectively. However, the substrate affinities for both compounds are fairly high (*K*_m_ = 159.9 ± 41.1 and 72.5 ± 15.2 μM, **Table 1**) and probably also preclude any *in situ* relevance of these *in vitro* data. The mass spectrum of P07 showed a molecular ion peak at *m/z* 207 [M-H]^+^ (**Supplementary Figure S4C**), which agrees with a molecular formula of C_11_H_10_O_4_, indicating 16 mass units more than the mass of the substrate, 3-methyl-7-methoxycoumarin. Thus, in accordance with incubation with CYP71AZ4, the product P07 was hypothesized to possess an additional oxygen atom. Subsequent NMR analysis showed that P07 was 8-hydroxy-3-methyl-7-methoxycoumarin (**Supplementary Figure S5**).

**FIGURE 6:**
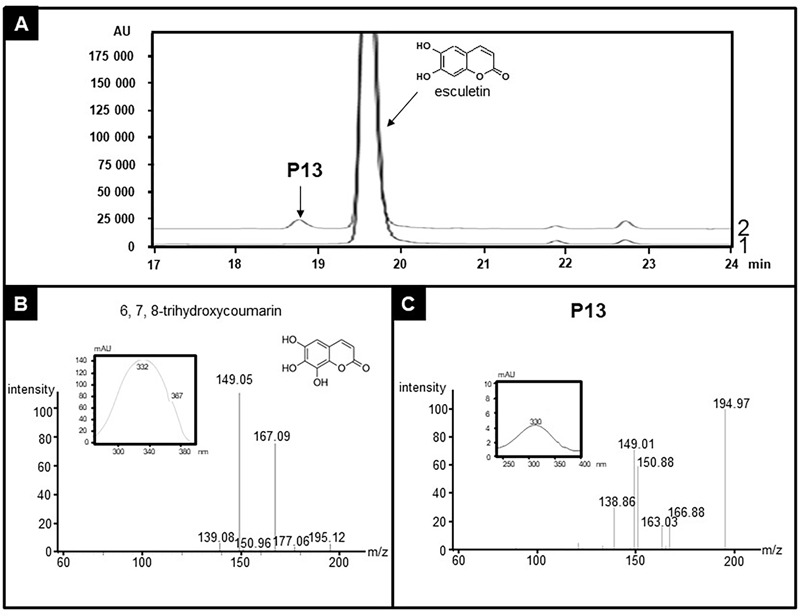
Identification of the product of esculetin metabolization by CYP71AZ3. Yeast microsomes expressing CYP71AZ3 convert esculetin to 6,7,8-trihydroxycoumarin (P13). **(A)** HPLC profile of enzyme assays containing esculetin, without (line 1) or with (line 2) NADPH, oxygen, and yeast microsomes expressing CYP71AZ3. **(B)** MS and absorption spectrum of standard 6,7,8-trihydroxycoumarin. **(C)** MS and absorption spectrum of P13.

Extended enzyme assays also disclosed some side activities for CYP71AZ1 and CYP71AZ6. In addition to xanthotoxin, both enzymes hydroxylated 7-methoxycoumarin with very poor efficiencies to 5-hydroxy-7-methoxycoumarin (P09 or P10, **Supplementary Figures S6A–E**). Furthermore, both enzymes also slowly metabolized 6-methoxycoumarin to products P11 and P12 (**Supplementary Figures S7A,C**). We were not able to measure the catalytic parameters for this set of experiments with 7-methoxycoumarin and 6-methoxycoumarin as substrates. The products share their MS profiles (**Supplementary Figures S7B,D**) but could not be identified by NMR because of their limited availability. Finally, the screening also revealed a substrate for CYP71AZ3, the gene that did not respond to the wounding of *P. sativa* plants. This enzyme exclusively accepted esculetin among all the compounds included in the assays (**Figure 6A**) and formed (**Table 1**) 6,7,8-trihydroxycoumarin with a poor turnover rate (*K*_m_ = 248.6 ± 51.9 μM). This product was identified by absorption spectroscopy as well as MS analyses (**Figures 6B,C** and **Supplementary Figure S8**). The mass spectrum of P13 showed a molecular ion peak at *m/z* 195 [M-H]^+^ (**Figure 6C**), which is in agreement with a molecular formula of C_9_H_6_O_5_, indicating 16 mass units more than the mass of the substrate, esculetin. Thus, in accordance with incubation with CYP71AZ3, the product P13 was hypothesized to possess an additional oxygen atom. The structure of 6,7,8-trihydroxycoumarin was further confirmed by NMR (**Supplementary Figures S8, S9**).

The enzymatic and analytical data underline the significance of the CYP71AZ subfamily of enzymes for the overall synthesis of coumarins and FCs (**Table 1**) and adds several pieces to the FC pathway puzzle. Surprisingly, CYP71AZ4 displayed a broad range of substrate specificities *in vitro* and may be involved in multiple branches of the coumarin biosynthesis *in situ*. This result is noteworthy because the other CYP71AZs catalyze more well-defined steps in these pathways.

### Identification of Prominent Regions in the Catalytic Sites of CYP71AZ4 and CYP71AZ3

**FIGURE 7:**
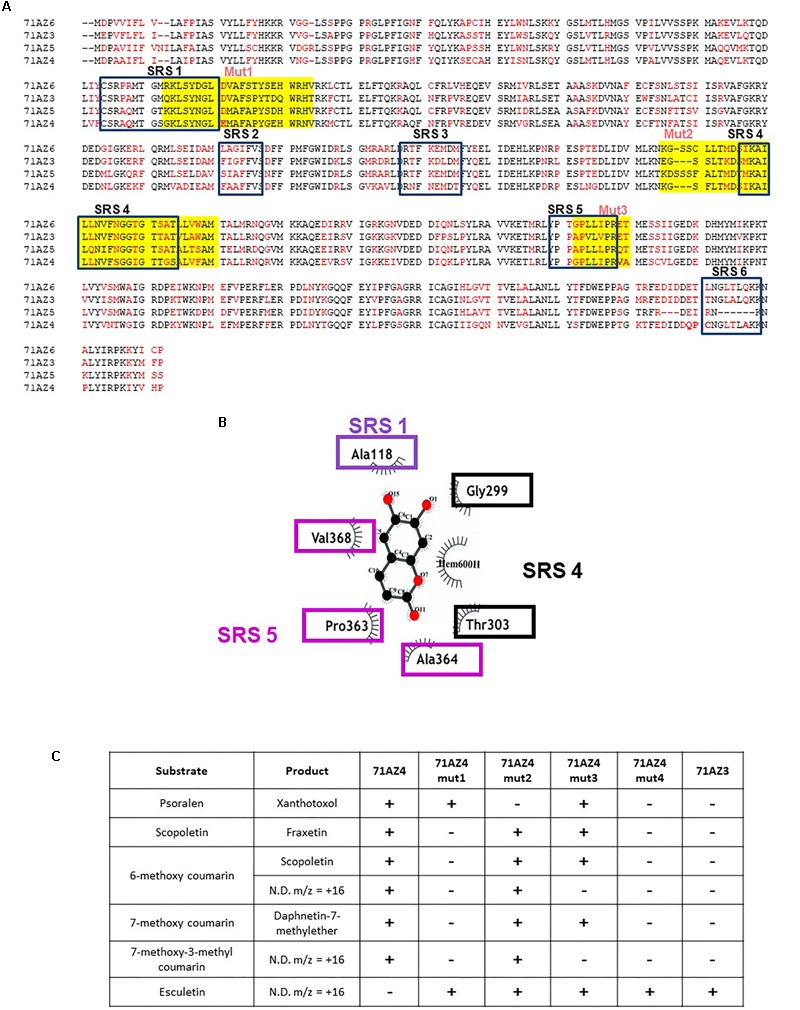
Sequence alignment of *P. sativa* CYP71AZ1, CYP71AZ3, CYP71A74, and CYP71AZ6 and construction of the CYP71AZ4 mutants. **(A)** Alignment of the four peptide sequences. Red amino acids are divergent among the four proteins. SRSs are substrate recognition sites (Rupasinghe et al., 2003). Yellow boxes highlight the three regions that were swapped for the mutagenesis. **(B)** Identification of the amino acids involved in the interaction with esculetin as substrate. **(C)** Summary of the metabolization of various substrates by the CYP71AZ mutants. ND: not determined.

Alignments of the four complete CYP71AZ peptide sequences identified from parsnip display a moderate degree of conservation (**Figure 7**). To better understand the structure/activity relationship of these enzymes, we compared the highly specific for coumarin enzyme CYP71AZ3 and the broadly specific for coumarin/FC enzyme CYP71AZ4. An *in silico* model of CYP71AZ3 was constructed with esculetin fitted in the active site. This model was superimposed with sequences of the other CYP71AZs using the “Kpax” software (Ritchie et al., 2012) and led us to identify several regions that clearly differ in their active site conformations. These regions include substrate recognition sites (SRSs) (Rupasinghe et al., 2003) SRS1, SRS4 (alpha helix), and SRS5 (beta turn) (**Figure 7B**). Consequently, three CYP71AZ4 mutants were constructed (mut1-3, **Figure 7C**) to corroborate the involvement of these regions in the differential substrate specificities of CYP71AZ4 and CYP71AZ3. In these mutants, either one of these regions of wild-type CYP71AZ4 was replaced by the corresponding CYP71AZ3 peptide sequences (Nucleotide sequence 318-386 for mutant 1; 837-936 for mutant 2; and 1089-1115 for mutant 3). A fourth mutant was also constructed, hosting the three modifications in a single protein. The enzymatic characterization of the mutants expressed in yeast revealed four different substrate specificities (**Figure 7C**). The swapping of the mut2 sequence most likely affected the volume of the active site pocket since psoralen was no longer metabolized. The replacement of SRS1 (mut1) narrowed the substrate specificity to solely target esculetin (in addition to psoralen), which is not even a substrate for wild-type CYP71AZ4. The exchange of SRS5 (mut3) caused an intermediate type of substrate specificity. Finally, the simultaneous swapping of the three regions (mut4) resulted in a mutant CYP71AZ4 whose specificity was identical to that of CYP71AZ3 (**Figure 7C**). In summary, the data provided unequivocal evidence that the SRS regions of CYP71AZs are intimately involved in the specificities of these enzymes and confirm the relevance commonly assigned to SRSs in cytochrome P450s.

## Discussion

The adaptation of plants to changed environmental conditions often requires the creation of modified or novel enzymatic capabilities, and the P450 superfamily is particularly susceptible to such evolutionary processes as confirmed by the vast array of reactions catalyzed by this multigene family (Mizutani and Ohta, 2010; Mizutani and Sato, 2012). This adaptation is illustrated, for example, by the accumulation of FCs in umbelliferous plants infested with herbivorous insects (Berenbaum, 2001, 2002; Jogesh et al., 2015). The production and variation of FCs under these conditions were interpreted as the outcome of an “arms race” between plants and insects. The biosynthetic pathway leading to FCs has been studied for many years in Apiaceae and was shown in the 1980s to involve the sequential action of several P450s (Hamerski and Matern, 1988a,b). The first gene of this enzyme category, CYP71AJ1, was identified in 2007 (Larbat et al., 2007).

An intimate cooperation of P450s from wheat or maize has been demonstrated (Jonczyk et al., 2008), converting indole-3-glycerol phosphate (from the L-tryptophan pathway) to benzoxazinones by several variants of a single P450 subfamily (CYP71D). Such variants have likely evolved from one ancestor. The same mechanism was suspected to lead to the synthesis of FCs. This hypothesis initially gained support by the functional characterization of CYP71AJ4 (Larbat et al., 2009) catalyzing the synthesis of angelicin which is the analogous reaction in the pathway branching to angular FCs (**Figure 2**). Nevertheless, further studies of diversification revealed the presence of CYP71AJ paralogs in some Apiaceae species that do not produce FCs (Dueholm et al., 2015). In addition, no other CYP71AJs were related to the synthesis of FCs. With the discovery of the blooming CYP71AZ P450 subfamily, the hypothesis that only a few number of P450 families are specialized in the synthesis of FCs and coumarins had been revived. Since simple coumarins are abundantly present in spermatophytes, and FCs are present in only a few of them, the plants may have gained new CYP71AZ functionalities during evolution concomitant with or after those of the CYP71AJ family.

The study and functional characterization of five different CYP71AZs reported here provides evidence that each of them has its own evolutionary history. CYP71AZ3 is not overexpressed upon wounding of *P. sativa* plants. Our experiments show that this enzyme does not metabolize FCs but rather accepts a coumarin as substrate, although the catalytic properties determined *in vitro* suggest that esculetin is not the physiological substrate. Therefore, to identify the enzyme real substrate, the screening must be extended to other coumarins. CYP71AZ1 and 6 are very close to each other and have a restricted substrate specificity dedicated to the synthesis of an FC, which might reflect that they were selected with the appearance of the pathway and are now almost specialized for the synthesis of xanthotoxol. These reactions are catalyzed with reasonable *K*_m_ values. The evolution of this cytochrome P450 subfamily might therefore be close to that of the CYP71AJ subfamily. We can assume that the CYP71AZ and CYP71AJ P450 subfamily were originally involved in the synthesis of some common metabolites and evolved to be able to participate to the synthesis of FCs.

The emergence of the branching pathway to linear and angular FCs with CYP71AJs as the pivotal enzymes (Larbat et al., 2007, 2009; Dueholm et al., 2015) was a major breakthrough for the defense of these plants. CYP71AZ4 might thus be allocated to an intermediate stage to convert not only simple coumarins but also FCs. Regarding coumarins, CYP71AZ4 was shown to convert 6-methoxycoumarin to scopoletin *in vitro* at a physiological acceptable *K*_m_ (9.5 μM), and scopoletin can be further converted to fraxetin. However, this second reaction required an extraordinary high scopoletin concentration (*K*_m_ = 762.2 μM). CYP71AZ4 also accepts psoralen as a substrate (*K*_m_ = 11.3 μM, **Table 1**). Since the *K*_m_ values are close to each other and in an acceptable physiological range, CYP71AZ4 may be an enzyme on its way to becoming specialized in the evolutionary scheme.

Finally, CYP71AZ5 has no assigned function. The fact that this coding sequence is not transcribed and could not be cloned under our experimental conditions could reflect a pseudogenization or a gene duplication where the neofunctionalization has not yet occurred. From the evolutionary point of view, this gene might be close to CYP71AZ4, which is consistent with the phylogenetic tree. Thus, gene duplication and neofunctionalization appear to have occurred at several stages of the evolution of the CYP71AZ subfamily of genes and in close association with the evolution of CYP71AJ sequences. This evolution can be mimicked with targeted mutagenesis. Our sequence-swapping experiments showed that modifications required for modifying one enzyme into another are not limited to simple amino acid changes (mut1-3) but also involve modification of the tertiary structure of the protein (mut 4). Such a directed evolutionary strategy could be used to develop enzymes with new functionalities, as it has already been described elsewhere (Park et al., 2009).

A screening of various databases such as Onekp^5^, The European Nucleotide Archive^6^, phytometasyn^7^ using CYP71AZ1 as target makes evidence that only two plant families harbored paralogous genes with significantly high enough homologies to belong to the same P450 subfamily. Intriguingly, no CYP71AZ could be identified in other plants producing FCs such as Rutaceae (citrus for example) or Moraceae (Ficus for example). Although we could not test all the plants belonging to these plant species because of the absence of available databases, it might be possible that the synthesis of these molecules in other plants is realized by other genes and is in favor of a convergent evolution as suggested previously (Berenbaum, 1983; Bourgaud et al., 2014, Munakata et al., 2016).

All the CYP71AZ paralogous genes, highlighted in our transcriptomic screening, were identified in Apiaceae except CYP71AZ10, which was identified in *C. racemosa*. Interestingly, Moon et al. (2011) shows that this plant is able to produce isoimperatorin. An additional search done through the Dictionary of Natural Products^8^ also showed the presence some molecules which are structurally related to FC such as (norvisnagin: 4-hydroxy-7-methylfuro[3,2-*g*]chromen-5-one (chromene) and angelicain: 4-hydroxy-7-(hydroxymethyl)-2-(2-hydroxypropan-2-yl)-2,3-dihydrofuro[3,2-*g*]chromen-5-one). It might be possible that CYP71AZ10 could be involved in the synthesis of these molecules. A deeper analysis of new transcriptomic data will probably help, in the near future to get more tracks for identifying the function of this enzyme or other new paralogues of CYP71AZ.

To identify the function of other CYP71AZ, the emergence and publication of many metabolomics studies will help to identify potential substrates. We show that CYP71AZ4 was able to metabolize a few coumarins. Among the molecule tested, only two of them could be identified in parsnip extracts (fraxetin and osthol, **Supplementary Figure S1**). These result makes evidence that this enzyme is obviously not highly specific. An extensive screening should be realized based on phytochemicals studies. For example, very few reports showed the presence of several coumarins in the roots of *T. garganica* and are present in very low amounts. Among these molecules, various authors described scopoletin and 6-methoxy-7-geranyloxycoumarin (Larsen and Sandberg, 1970; Rubal et al., 2007). CYP71AZ2 may be involved in the synthesis of this coumarin.

Plants produce a plethora of metabolites that are required for their growth and propagation as well as for regulatory needs and adaptation to environmental conditions. Among the 370,000 predicted vascular plants, only a small part has been studied at the chemical level (Lughadha et al., 2016). According to the Royal Botanical Kew Garden, 28,000 plants are useful for medicines and so far 300,000 specialized metabolites were identified throughout the plant diversity (Hubert et al., 2017). Here, we provide a new example of the importance of cytochrome P450s, which have already been identified to be deeply involved in this diversification (Nelson and Werck-Reichhart, 2011), and suggest how they can be involved in the appearance of new molecules to meet further challenges for an ecological function (Pichersky and Gang, 2000; Lewinsohn and Gijzen, 2009).

## Author Contributions

CK and SR cloned the genes (CYP71AZ3, 4, 6, and AZ4mut1-4), expressed all the proteins in yeast and realized the kinetic characterization. SK-T did the *A. majus* cell cultures and identified and isolated CYP71AZ1. GG did the induction of parsnip and the RT-qPCR experiments for CYP71AZs and CYP71AJ3. BS did the NMR analyses. JG did the LC/manuscript analyses. AO did the biochemical analyses and wrote the manuscript. DR performed the modeling and Kpax experiments and edited the manuscript. UM led the work done on the *A. majus* cell cultures and edited the manuscript. FB and AH led the work done on the CYP71AZ characterization. AH led the whole project and wrote the manuscript.

## Conflict of Interest Statement

The authors declare that the research was conducted in the absence of any commercial or financial relationships that could be construed as a potential conflict of interest.

## Footnotes

1 https://www.sigmaaldrich.com/

2 https://www.thermofisher.com/

3 https://www.clontech.com/

4 http://apiaceae.njau.edu.cn

5 http://onekp.com

6 https://www.ebi.ac.uk/ena

7 https://bioinformatics.tugraz.at/phytometasyn

8 http://dnp.chemnetbase.com

## Supplementary Material

FIGURE S1 Name, molecular weight, chemical structure, and code access number of the molecules used for the *in vitro* screening of CYP71AZs.

FIGURE S2 Identification of the products of 6-methoxycoumarin metabolization **(A)** by CYP71AZ4. Yeast microsomes expressing CYP71AZ4 convert 6-methoxycoumarin to P04 and P05. **(A)** HPLC profile of enzyme assays containing 6-methoxycoumarin, without (line 1) or with (line 2) NADPH, oxygen, and yeast microsomes expressing CYP71AZ4. **(B)** MS and absorption spectrum of standard scopoletin. **(C)** MS and absorption spectrum of P04. **(D)** MS and absorption spectrum of P05.

FIGURE S3 Identification of the product of scopoletin metabolization by CYP71AZ4. Yeast microsomes expressing CYP71AZ4 convert scopoletin to P06. **(A)** HPLC profile of enzyme assays containing scopoletin, without (line 1) or with (line 2) NADPH, oxygen, and yeast microsomes expressing CYP71AZ4. **(B)** MS and absorption spectrum of standard Fraxetin. **(C)** MS and absorption spectrum of P06.

FIGURE S4 Identification of the products of 3-methyl-7-methoxycoumarin **(A)** and 7-methoxycoumarin **(B)** metabolization by CYP71AZ4. Yeast microsomes expressing CYP71AZ4 convert 3-methyl-7-methoxycoumarin to P07 and 7-methoxycoumarin to P08. **(A)** HPLC profile of enzyme assays containing 3-methyl-7-methoxycoumarin, NADPH, oxygen, and yeast microsomes expressing CYP71AZ4. **(B)** HPLC profile of enzyme assays containing 7-methoxycoumarin, without (line 1) or with (line 2) NADPH, oxygen, and yeast microsomes expressing CYP71AZ4. **(C)** MS and absorption spectrum of P07. **(D)** MS and absorption spectrum of standard daphnetin 7-methyl ether. **(E)** MS and absorption spectrum of P08

FIGURE S5 ^1^H NMR spectra (700 MHz, acetone-*d*_6_) of a sample containing 8-hydroxy-3-methyl-7-methoxy coumarin (P07). **(A)** Full spectrum. **(B)** Extended region showing aromatic and double bond signals of P07 (marked with green arrows). **(C)** Extended region showing the methoxy signal of P07 (marked with green arrow). ^1^H NMR (700 MHz, acetone-d_6_): δ 7.03 (1H, J = 8.2 Hz, H-5), 6.98 (1H, d, J = 8.2 Hz, H-6), 7.65 (1H, s, H-4), 3.92 (1H, s, 7-OCH_3_), signal of 3-CH_3_ not detected due to overlap with matrix signals. ^13^C NMR (175 MHz, acetone-d_6_, signal extracted from HSQC spectrum): δ 56.6 (7-OCH_3_) HSQC cross signals of other carbon atoms were not detected due to limited amount of material. The ^1^H NMR spectrum of P07 (700 MHz, acetone-*d*_6_) displays doublets (*J* = 8.2 Hz) of an AB spin system comprising H-5 (δ 7.03) and H-6 (δ 6.98) of the aromatic ring in this figure. The proximity of H-5 and H-6 was confirmed by a strong roof effect of the two ^1^H NMR signals and a correlation signal in the ^1^H,^1^H COSY spectrum. The singlet at δ 3.92 (3H) and an HSQC correlation signal δ_H_ 3.92/δ_C_ 56.6 are assignable to the methoxy group (7-OCH_3_). The signal of the methyl group protons at C-3 remained obscured due to overlap with matrix signals. Since the presence of the H-5/H-6 AB spin system ruled out hydroxylation at C-5 and C-6, positions 4 and 8 remained possibilities for the new hydroxyl group. Thus, the singlet of the isolated aromatic proton resonating at δ 7.65 (1H) had to be assigned to either to H-4 or H-8. A proton at C-8, corresponding to hydroxylation at C-4, could be disregarded because an *m*-coupling with H-6 was missing and the chemical shift of H-8 would be expected near δ 6.5. The latter value is largely different from the observed chemical shift of δ 7.65, which is consistent with the value expected for H-4. Finally, hydroxylation at the 2-methyl group could also be excluded because the ^1^H NMR signal of a prochiral hydroxymethylene group with an expected chemical shift of approximately δ 4.5 was absent from the spectrum. From these data, P07 was identified as 8-hydroxy-3-methyl-7-methoxycoumarin

FIGURE S6 Identification of the products of 7-methoxycoumarin metabolization by CYP71AZ1 **(A and D)** and CYP71AZ6 **(B and E)**. Yeast microsomes expressing CYP71AZ1 and CYP71AZ6 convert 7-methoxycoumarin to P09 and P10, respectively. **(A)** HPLC profile of enzyme assays containing 7-methoxycoumarin, without (line 1) or with (line 2) NADPH, oxygen, and yeast microsomes expressing CYP71AZ1. **(B)** HPLC profile of enzyme assays containing 7-methoxycoumarin, without (line 1) or with (line 2) NADPH, oxygen, and yeast microsomes expressing CYP71AZ1. **(C)** MS and absorption spectrum of 5-hydroxy-7-methoxycoumarin. **(D)** MS and absorption spectrum of P09. **(E)** MS and absorption spectrum of P10.

FIGURE S7 Identification of the products of 6-methoxycoumarin metabolization by CYP71AZ1 **(A and B)** and CYP71AZ6 **(C and D)**. Yeast microsomes expressing CYP71AZ1 and CYP71AZ6 convert 6-methoxycoumarin to P11 and P12, respectively. **(A)** HPLC profile of enzyme assays containing 6-methoxycoumarin, without (line 1) or with (line 2) NADPH, oxygen, and yeast microsomes expressing CYP71AZ1. **(B)** MS and absorption spectrum of P11. **(C)** HPLC profile of enzyme assays containing 6-methoxycoumarin, without (line 1) or with (line 2) NADPH, oxygen, and yeast microsomes expressing CYP71AZ6. **(D)** MS and absorption spectrum of P12.

FIGURE S8 ^1^H NMR spectra (700 MHz, MeOH-*d*_4_) of a sample containing 6,7,8-trihydroxycoumarin (P13). **(A)** Full spectrum. **(B)** Extended region showing signals of P13 (marked with green arrows). ^1^H NMR (700 MHz, MeOH-*d*_4_): δ 7.86 (1H, J = 8.2 Hz, H-4), 6.23 (1H, d, J = 8.2 Hz, H-3), 6.82 (1H, s, H-5). ^13^C NMR (175 MHz, MeOH-d_4_, signals extracted from HSQC spectrum): δ 112.6 (C-4), 146.0 (C-5), 103.6 (C-6). The ^1^H NMR spectrum of P13 (700 MHz, MeOH-*d*_4_) showed two doublets (at δ 7.86 and 6.23), which according to their chemical shifts and coupling constant of *J* = 8.2 Hz were assigned to the *Z*-configured double bond protons H-4 and H-5. A ^1^H,^1^H-COSY spectrum confirmed the AX spin system of these two protons. Hence, the new hydroxyl group cannot be located in the pyrone ring but must be attached to either position 5 or 8 of the aromatic ring. The signal of the remaining proton was detected at δ 7.68, and assignment to H-5 was achieved by comparing the chemical shift with reported data of 6,7,8-trihydroxycoumarin (Kayser and Kolodziej, 1995; Yasuda et al., 2006). Furthermore, the ^13^C chemical shifts of the protonated carbon atoms C-3, C-4, and C-5 were extracted from the HSQC spectrum of P13 (**Supplementary Figure S9**) and agreed with reported data (Kayser and Kolodziej, 1995; Yasuda et al., 2006) of 6,7,8-trihydroxycoumarin.

FIGURE S9 Partial HSQC NMR spectrum (700 MHz, MeOH-*d*_4_) of a sample containing 6,7,8-trihydroxycoumarin (P13). Top: ^1^H NMR spectrum. ^1^H NMR signals and ^1^H/^13^C correlation signals of P13 are marked with green arrows.

FIGURE S10 Sequences of the different CYP71AZs collected in databases.

TABLE S1 Sequence homologies between different CYP71AZs from *A. majus* (CYP71AZ1) and *P. sativa* (AZ3, AZ4, AZ5, and AZ6). Comparison of the full length sequences are in white squares). Comparison restricted to the six substrate recognition sites (SRS) are in gray squares.
