# Supplementary material for: The CYP71AZ P450 Subfamily: A Driving Factor for the Diversification of Coumarin Biosynthesis in Apiaceous Plants
Source: Front Plant Sci. 2018 Jun 19;9:820. doi: 10.3389/fpls.2018.00820 (PMC6018538; doi:10.3389/fpls.2018.00820)
Supplement: FIGURE S1 — Name, molecular weight, chemical structure, and code access number of the molecules used for the in vitro screening of CYP71AZs. [file Presentation_1.pptx]

## Slide 1
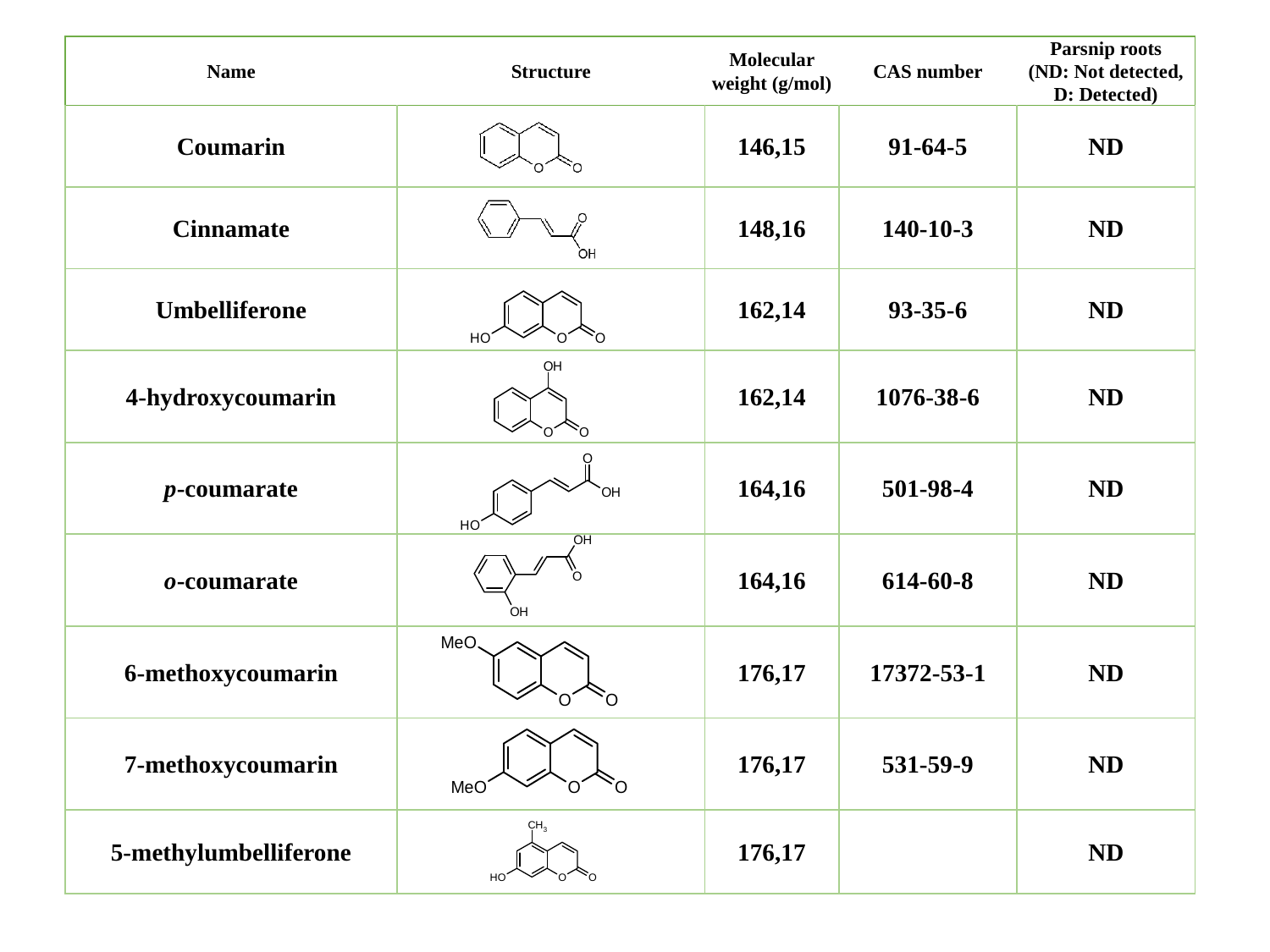

| Name | Structure | Molecular weight (g/mol) | CAS number | Parsnip roots (ND: Not detected, D: Detected) |
| --- | --- | --- | --- | --- |
| Coumarin | | 146,15 | 91-64-5 | ND |
| Cinnamate | | 148,16 | 140-10-3 | ND |
| Umbelliferone | | 162,14 | 93-35-6 | ND |
| 4-hydroxycoumarin | | 162,14 | 1076-38-6 | ND |
| p-coumarate | | 164,16 | 501-98-4 | ND |
| o-coumarate | | 164,16 | 614-60-8 | ND |
| 6-methoxycoumarin | | 176,17 | 17372-53-1 | ND |
| 7-methoxycoumarin | | 176,17 | 531-59-9 | ND |
| 5-methylumbelliferone | | 176,17 | | ND |

## Slide 2
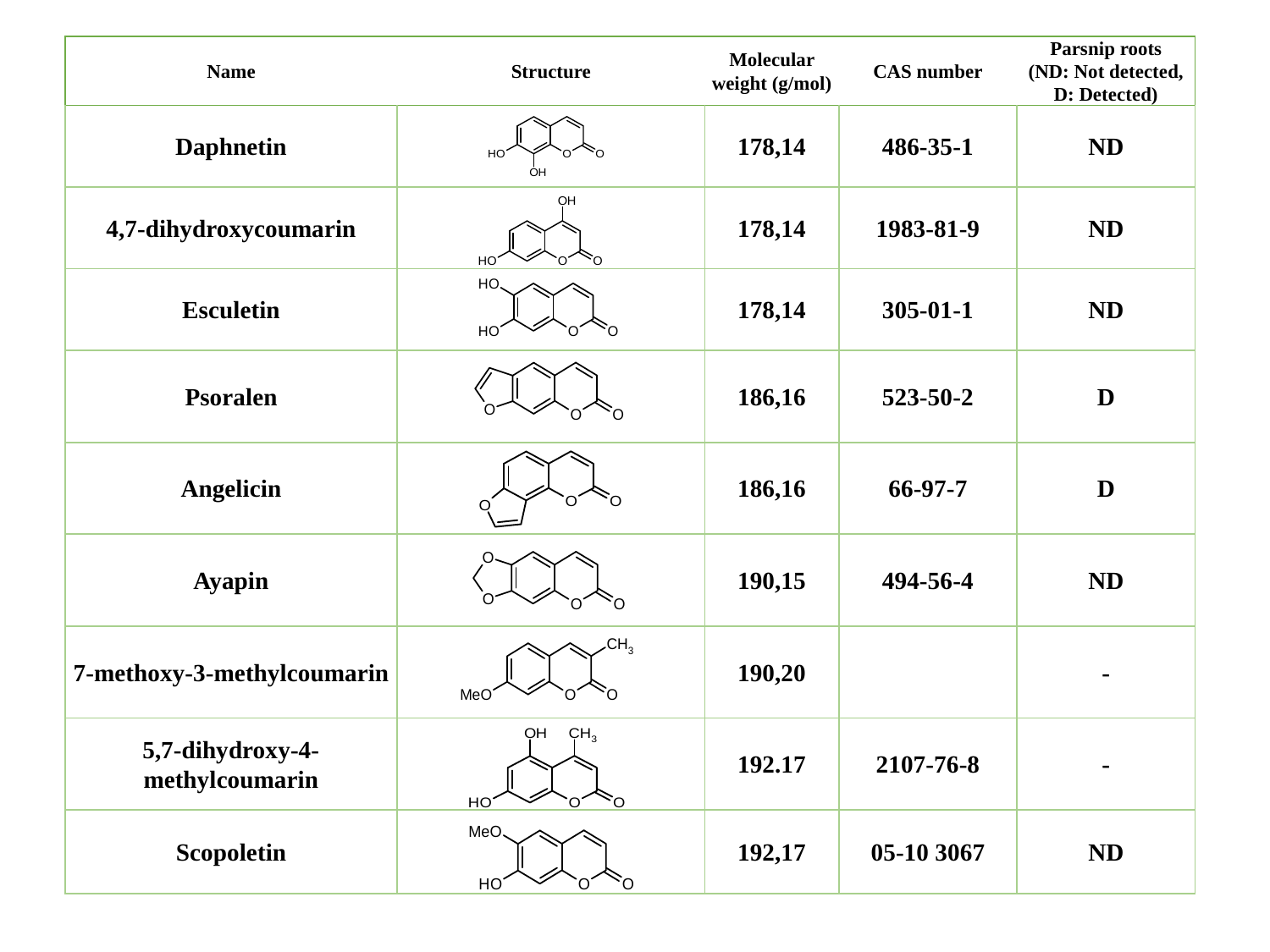

| Name | Structure | Molecular weight (g/mol) | CAS number | Parsnip roots (ND: Not detected, D: Detected) |
| --- | --- | --- | --- | --- |
| Daphnetin | | 178,14 | 486-35-1 | ND |
| 4,7-dihydroxycoumarin | | 178,14 | 1983-81-9 | ND |
| Esculetin | | 178,14 | 305-01-1 | ND |
| Psoralen | | 186,16 | 523-50-2 | D |
| Angelicin | | 186,16 | 66-97-7 | D |
| Ayapin | | 190,15 | 494-56-4 | ND |
| 7-methoxy-3-methylcoumarin | | 190,20 | | - |
| 5,7-dihydroxy-4-methylcoumarin | | 192.17 | 2107-76-8 | - |
| Scopoletin | | 192,17 | 05-10 3067 | ND |

## Slide 3
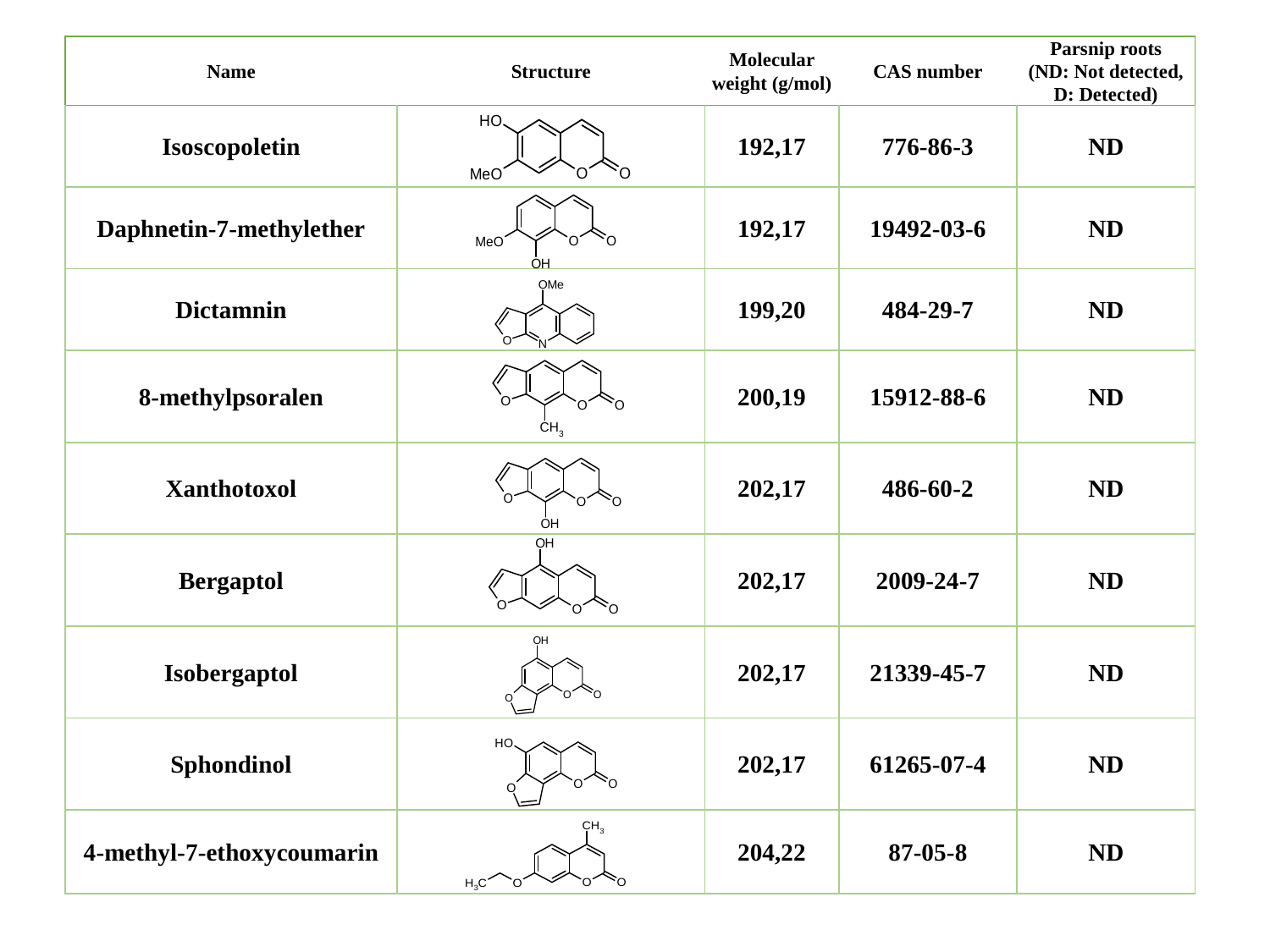

| Name | Structure | Molecular weight (g/mol) | CAS number | Parsnip roots (ND: Not detected, D: Detected) |
| --- | --- | --- | --- | --- |
| Isoscopoletin | | 192,17 | 776-86-3 | ND |
| Daphnetin-7-methylether | | 192,17 | 19492-03-6 | ND |
| Dictamnin | | 199,20 | 484-29-7 | ND |
| 8-methylpsoralen | | 200,19 | 15912-88-6 | ND |
| Xanthotoxol | | 202,17 | 486-60-2 | ND |
| Bergaptol | | 202,17 | 2009-24-7 | ND |
| Isobergaptol | | 202,17 | 21339-45-7 | ND |
| Sphondinol | | 202,17 | 61265-07-4 | ND |
| 4-methyl-7-ethoxycoumarin | | 204,22 | 87-05-8 | ND |

## Slide 4
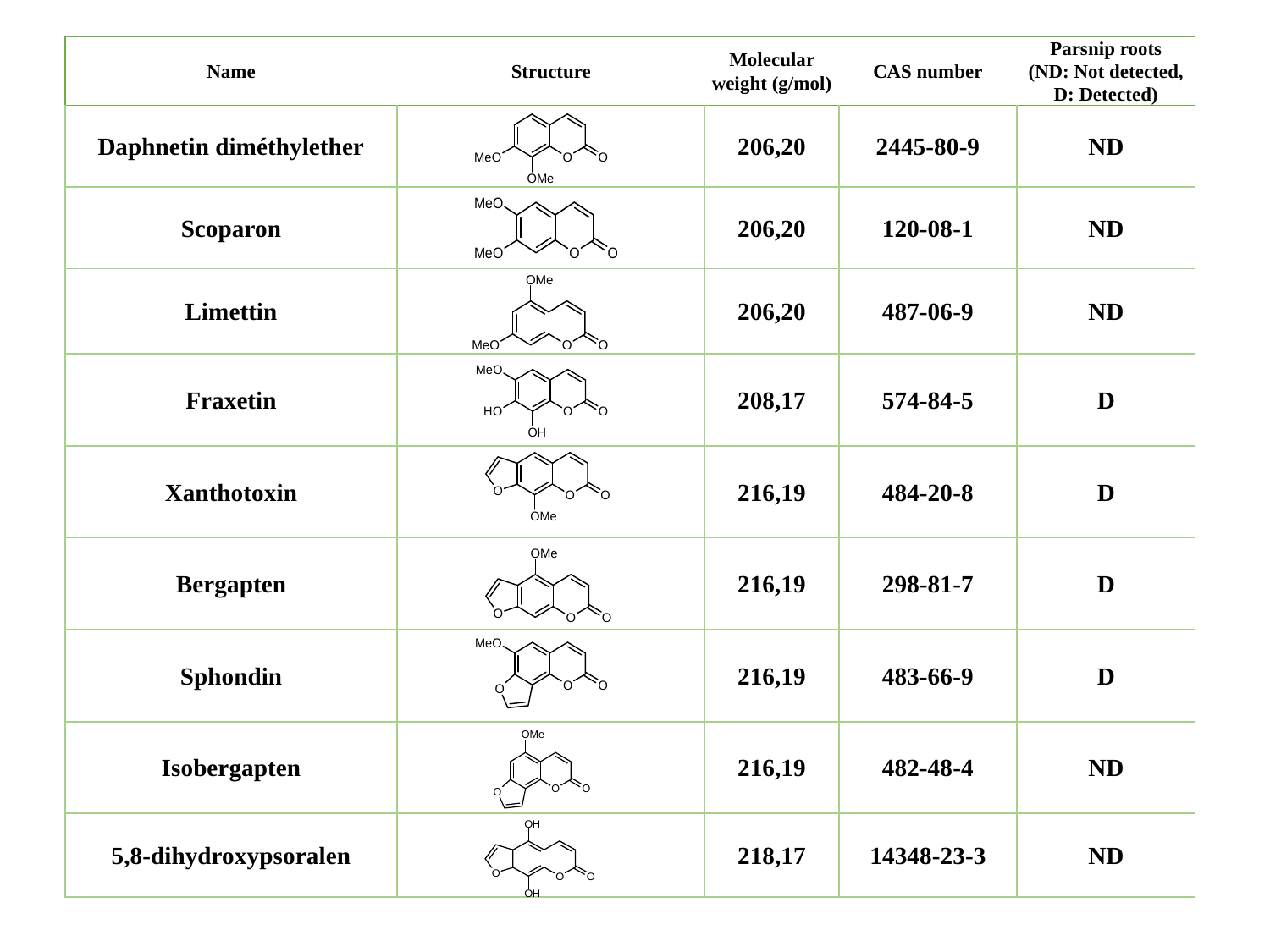

| Name | Structure | Molecular weight (g/mol) | CAS number | Parsnip roots (ND: Not detected, D: Detected) |
| --- | --- | --- | --- | --- |
| Daphnetin diméthylether | | 206,20 | 2445-80-9 | ND |
| Scoparon | | 206,20 | 120-08-1 | ND |
| Limettin | | 206,20 | 487-06-9 | ND |
| Fraxetin | | 208,17 | 574-84-5 | D |
| Xanthotoxin | | 216,19 | 484-20-8 | D |
| Bergapten | | 216,19 | 298-81-7 | D |
| Sphondin | | 216,19 | 483-66-9 | D |
| Isobergapten | | 216,19 | 482-48-4 | ND |
| 5,8-dihydroxypsoralen | | 218,17 | 14348-23-3 | ND |

## Slide 5
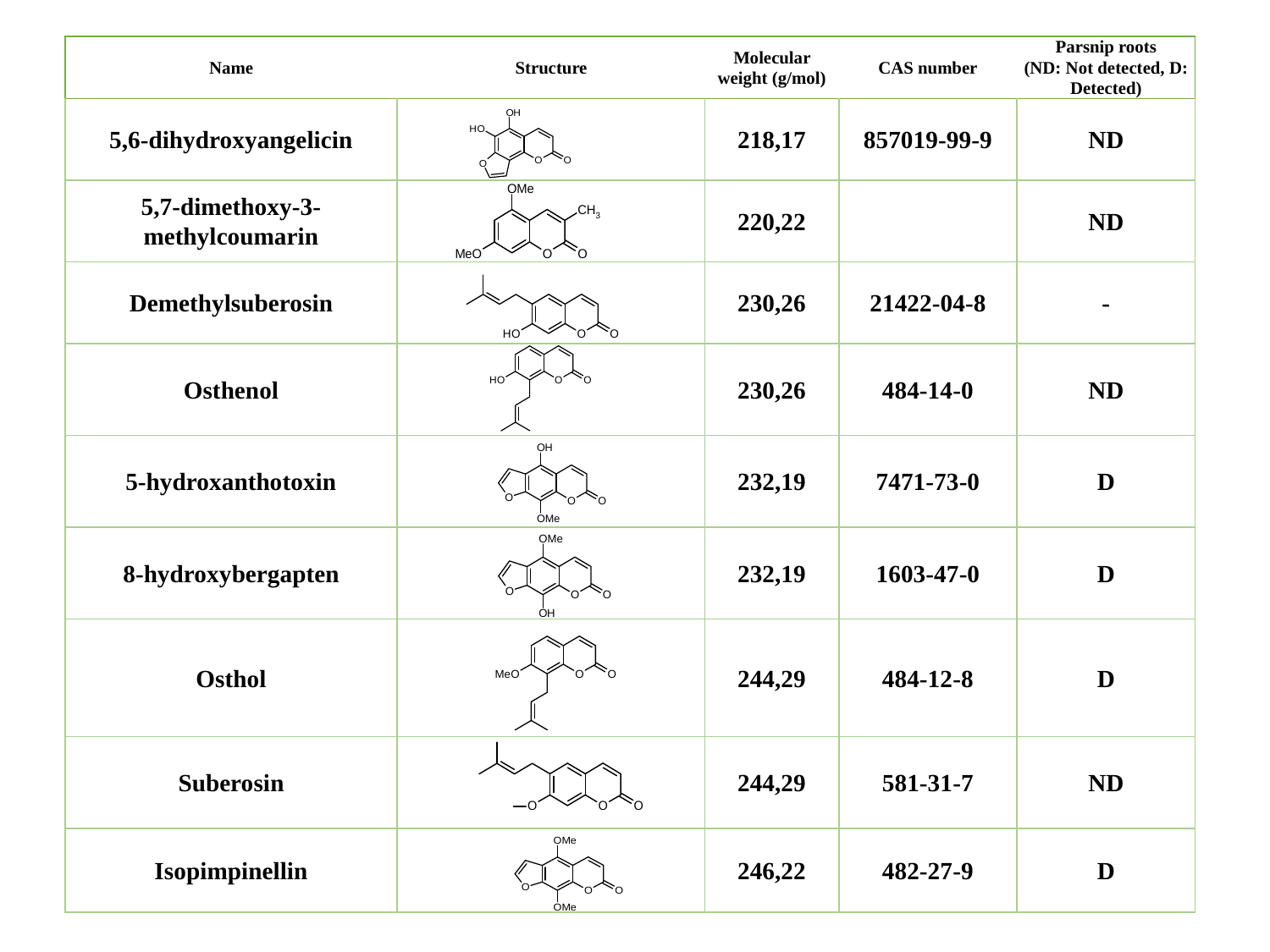

| Name | Structure | Molecular weight (g/mol) | CAS number | Parsnip roots (ND: Not detected, D: Detected) |
| --- | --- | --- | --- | --- |
| 5,6-dihydroxyangelicin | | 218,17 | 857019-99-9 | ND |
| 5,7-dimethoxy-3-methylcoumarin | | 220,22 | | ND |
| Demethylsuberosin | | 230,26 | 21422-04-8 | - |
| Osthenol | | 230,26 | 484-14-0 | ND |
| 5-hydroxanthotoxin | | 232,19 | 7471-73-0 | D |
| 8-hydroxybergapten | | 232,19 | 1603-47-0 | D |
| Osthol | | 244,29 | 484-12-8 | D |
| Suberosin | | 244,29 | 581-31-7 | ND |
| Isopimpinellin | | 246,22 | 482-27-9 | D |

## Slide 6
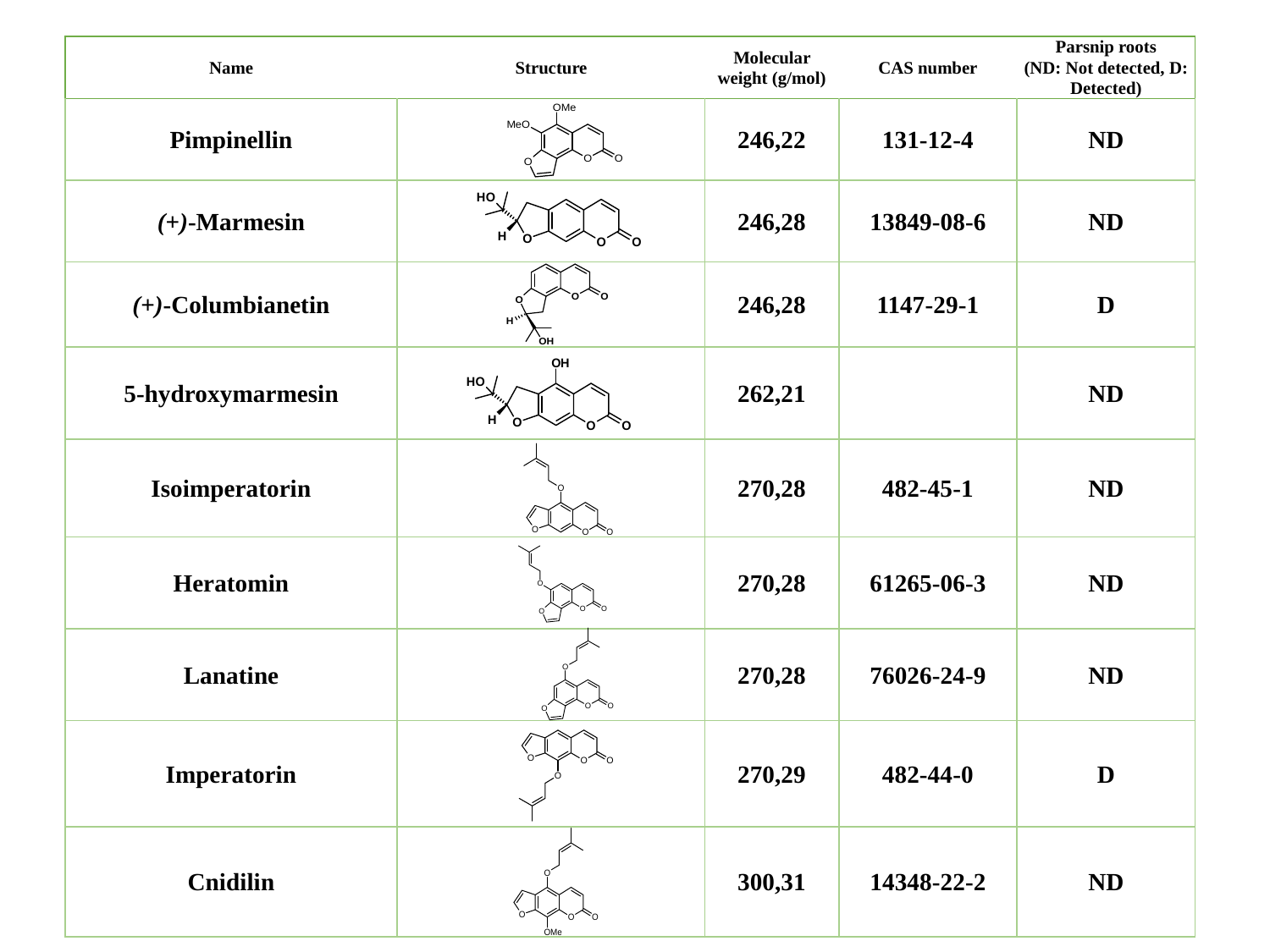

| Name | Structure | Molecular weight (g/mol) | CAS number | Parsnip roots (ND: Not detected, D: Detected) |
| --- | --- | --- | --- | --- |
| Pimpinellin | | 246,22 | 131-12-4 | ND |
| (+)-Marmesin | | 246,28 | 13849-08-6 | ND |
| (+)-Columbianetin | | 246,28 | 1147-29-1 | D |
| 5-hydroxymarmesin | | 262,21 | | ND |
| Isoimperatorin | | 270,28 | 482-45-1 | ND |
| Heratomin | | 270,28 | 61265-06-3 | ND |
| Lanatine | | 270,28 | 76026-24-9 | ND |
| Imperatorin | | 270,29 | 482-44-0 | D |
| Cnidilin | | 300,31 | 14348-22-2 | ND |

## Slide 7
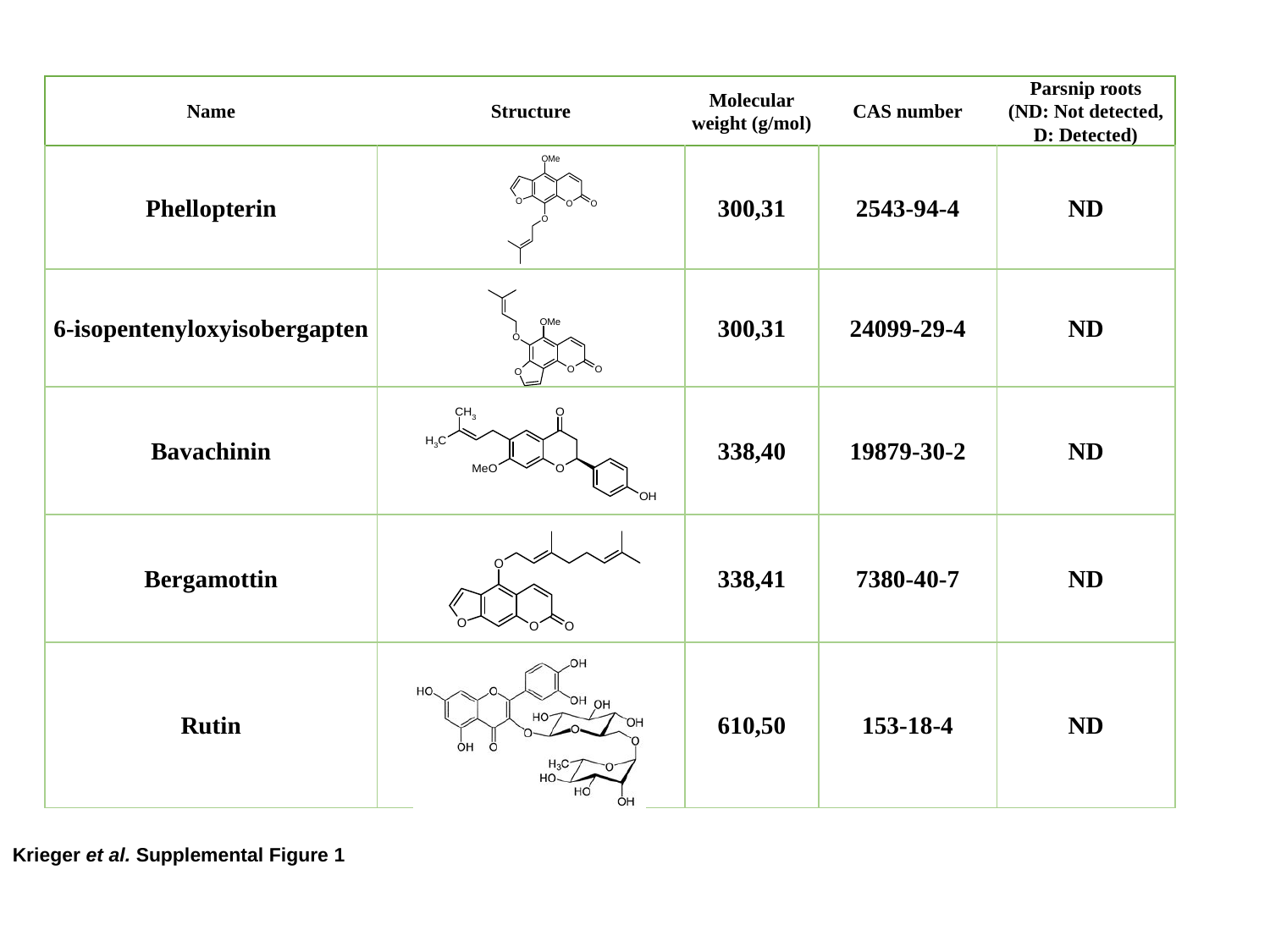

| Name | Structure | Molecular weight (g/mol) | CAS number | Parsnip roots (ND: Not detected, D: Detected) |
| --- | --- | --- | --- | --- |
| Phellopterin | | 300,31 | 2543-94-4 | ND |
| 6-isopentenyloxyisobergapten | | 300,31 | 24099-29-4 | ND |
| Bavachinin | | 338,40 | 19879-30-2 | ND |
| Bergamottin | | 338,41 | 7380-40-7 | ND |
| Rutin | | 610,50 | 153-18-4 | ND |
Krieger et al. Supplemental Figure 1

## Slide 8
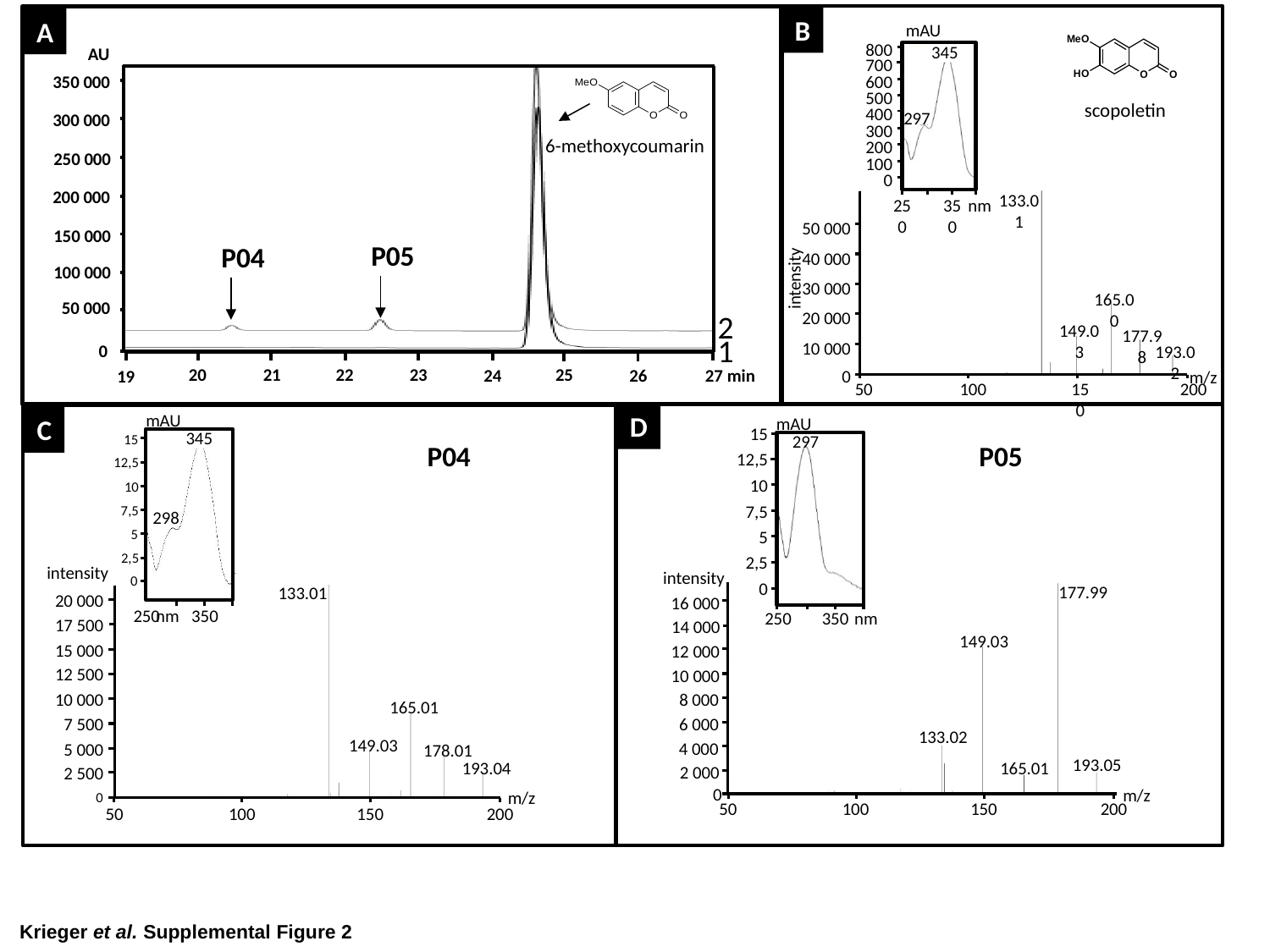

B
A
mAU
800
AU
350 000
300 000
6-methoxycoumarin
250 000
200 000
150 000
P05
P04
100 000
 50 000
2
1
0
25
20
21
22
23
24
min
27
26
19
345
700
600
500
scopoletin
400
297
300
200
100
0
133.01
250
350
nm
50 000
40 000
intensity
30 000
165.00
20 000
149.03
177.98
10 000
193.02
0
m/z
50
100
150
200
D
mAU
15
345
10
12,5
10
8
7,5
6
298
5
4
2,5
2
0
250
350
C
mAU
15
297
12,5
10
7,5
5
2,5
0
250
350
nm
P04
P05
intensity
intensity
177.99
16 000
14 000
149.03
12 000
10 000
8 000
6 000
133.02
m/z
4 000
193.05
165.01
2 000
0
m/z
50
100
150
200
133.01
20 000
nm
17 500
15 000
12 500
10 000
165.01
7 500
149.03
5 000
178.01
193.04
2 500
m/z
0
50
100
150
200
Krieger et al. Supplemental Figure 2

## Slide 9
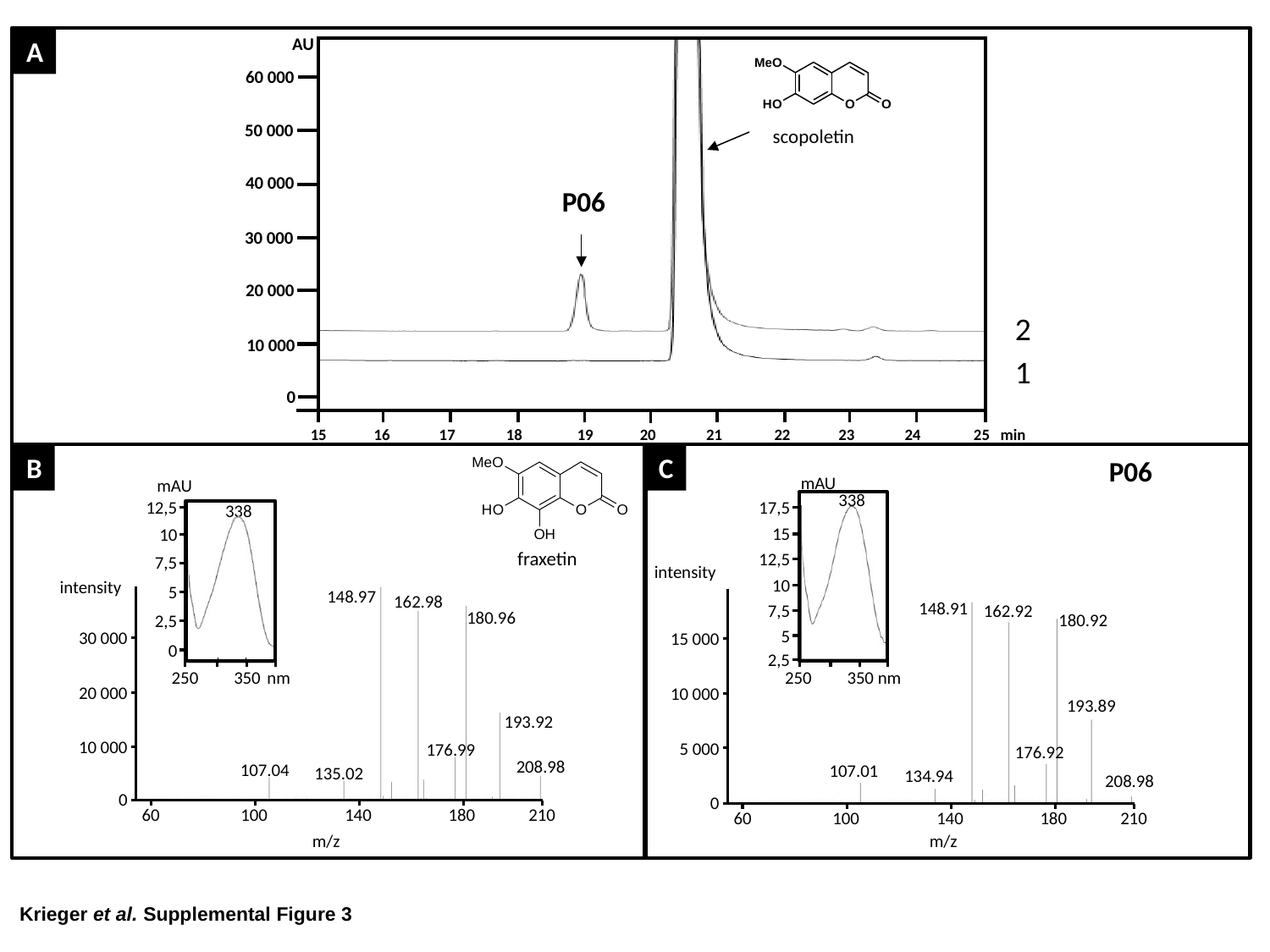

AU
A
60 000
50 000
scopoletin
40 000
P06
30 000
20 000
2
10 000
1
0
15
17
16
18
19
20
21
22
23
24
25
min
B
C
P06
mAU
338
17,5
15
12,5
10
7,5
5
2,5
250
350
nm
mAU
12,5
338
10
7,5
5
2,5
0
250
350
nm
fraxetin
intensity
148.91
162.92
180.92
15 000
10 000
193.89
5 000
176.92
107.01
134.94
208.98
0
60
100
140
180
210
m/z
intensity
148.97
162.98
180.96
30 000
20 000
193.92
10 000
176.99
208.98
107.04
135.02
0
60
100
140
180
210
m/z
Krieger et al. Supplemental Figure 3

## Slide 10
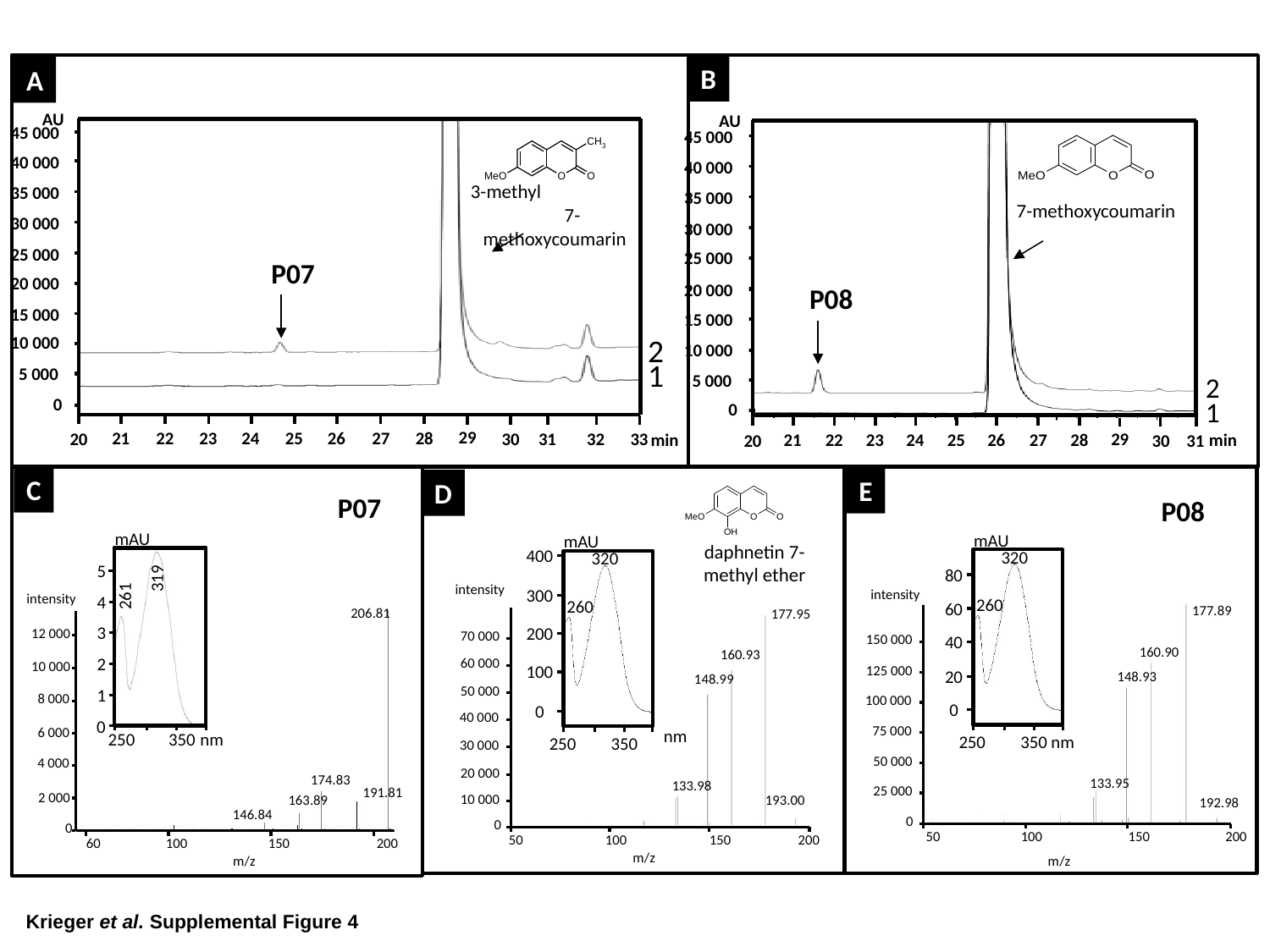

B
A
AU
45 000
40 000
3-methyl 7-methoxycoumarin
35 000
30 000
25 000
P07
20 000
P2
15 000
2
10 000
1
 5 000
0
29
21
22
23
24
25
26
27
28
30
31
32
33
20
min
AU
45 000
40 000
35 000
7-methoxycoumarin
30 000
25 000
20 000
P08
15 000
10 000
 5 000
2
1
0
29
21
22
23
24
25
26
27
28
min
30
31
20
C
E
D
P07
206.81
12 000
10 000
8 000
6 000
4 000
174.83
191.81
2 000
163.89
146.84
0
60
100
150
200
m/z
P08
mAU
5
319
4
261
3
2
1
0
250
350
nm
mAU
320
80
60
260
40
20
0
250
350
nm
mAU
400
320
300
260
200
100
0
nm
250
350
daphnetin 7-methyl ether
intensity
177.95
70 000
160.93
60 000
148.99
50 000
40 000
30 000
20 000
133.98
10 000
193.00
0
50
100
150
200
intensity
intensity
177.89
150 000
160.90
125 000
148.93
100 000
75 000
50 000
133.95
25 000
192.98
0
50
100
150
200
m/z
m/z
m/z
Krieger et al. Supplemental Figure 4

## Slide 11
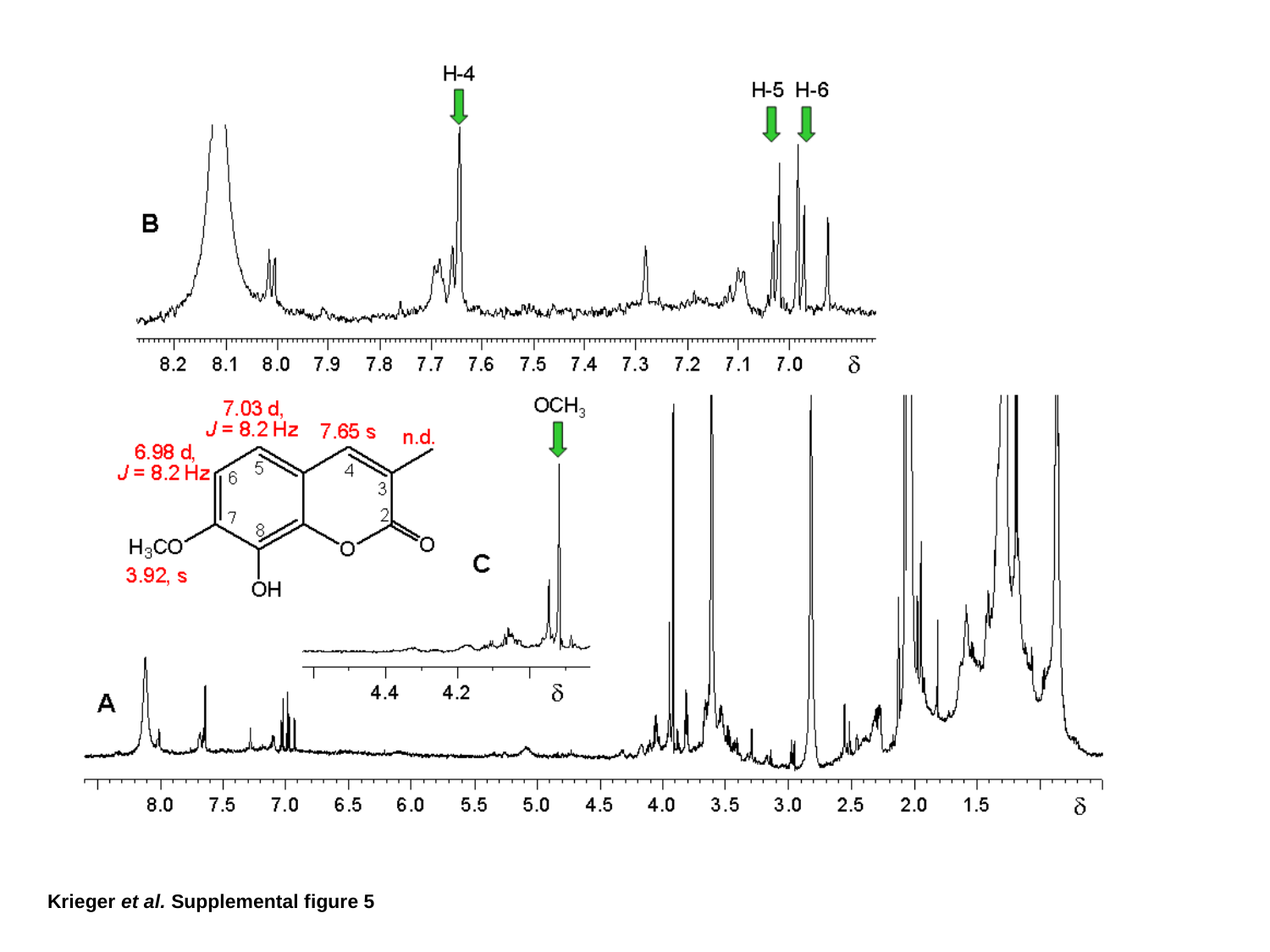

Krieger et al. Supplemental figure 5

## Slide 12
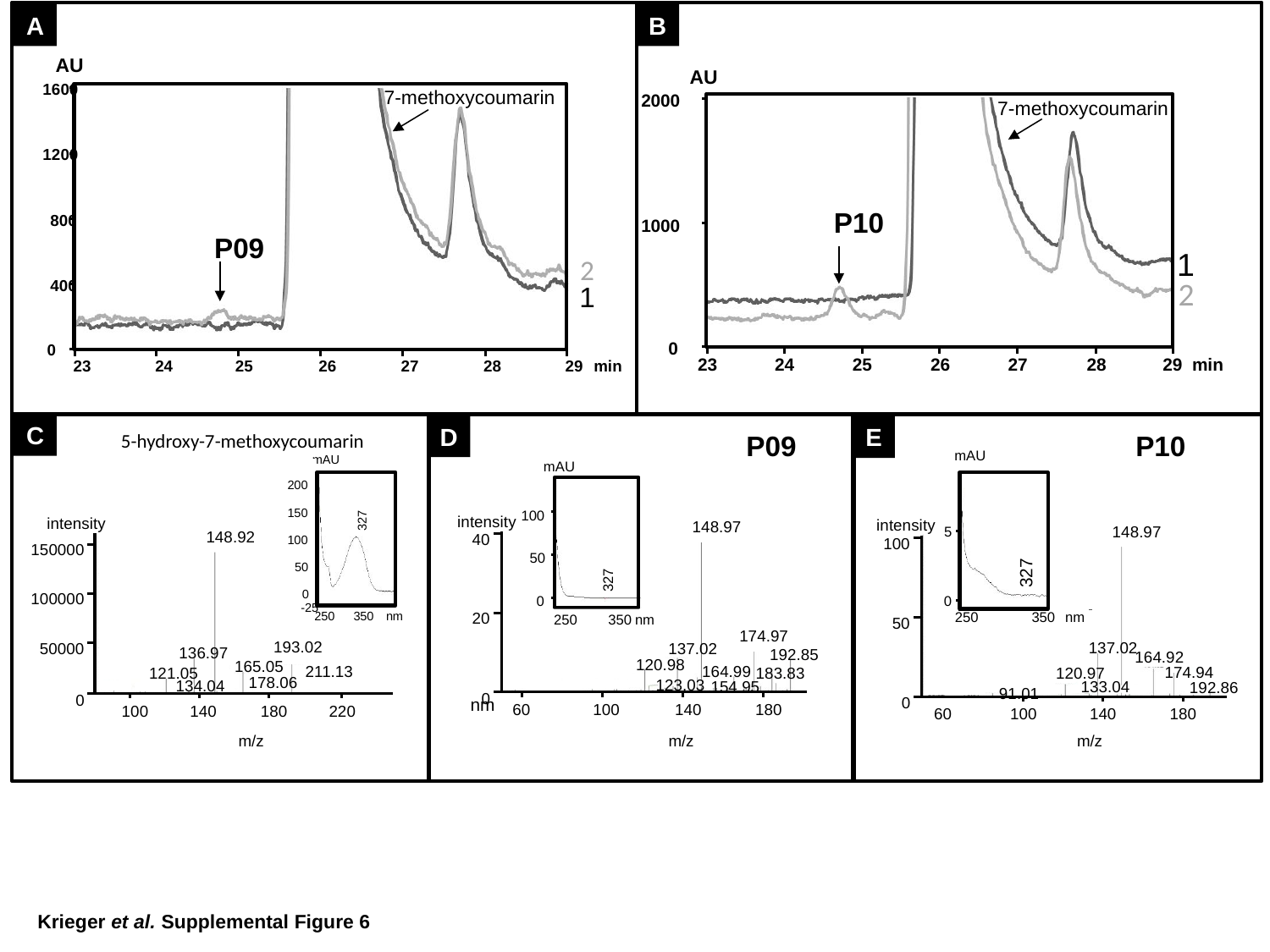

A
B
AU
AU
1600
7-methoxycoumarin
2000
7-methoxycoumarin
1200
P10
800
1000
P09
1
2
2
400
1
0
0
23
24
25
26
27
28
29
min
23
24
25
26
27
28
29
min
C
D
E
5-hydroxy-7-methoxycoumarin
P09
P10
mAU
mAU
mAU
200
150
100
intensity
intensity
327
intensity
148.97
148.97
5
148.92
40
100
100
150000
50
50
327
327
0
100000
0
0
-25
nm
250
350
nm
250
350
nm
20
nm
250
350
nm
50
174.97
193.02
137.02
137.02
50000
136.97
192.85
164.92
120.98
165.05
164.99
211.13
174.94
183.83
121.05
120.97
178.06
123.03
134.04
133.04
154.95
192.86
91.01
0
0
0
nm
60
100
140
180
100
140
180
220
60
100
140
180
m/z
m/z
m/z
Krieger et al. Supplemental Figure 6

## Slide 13
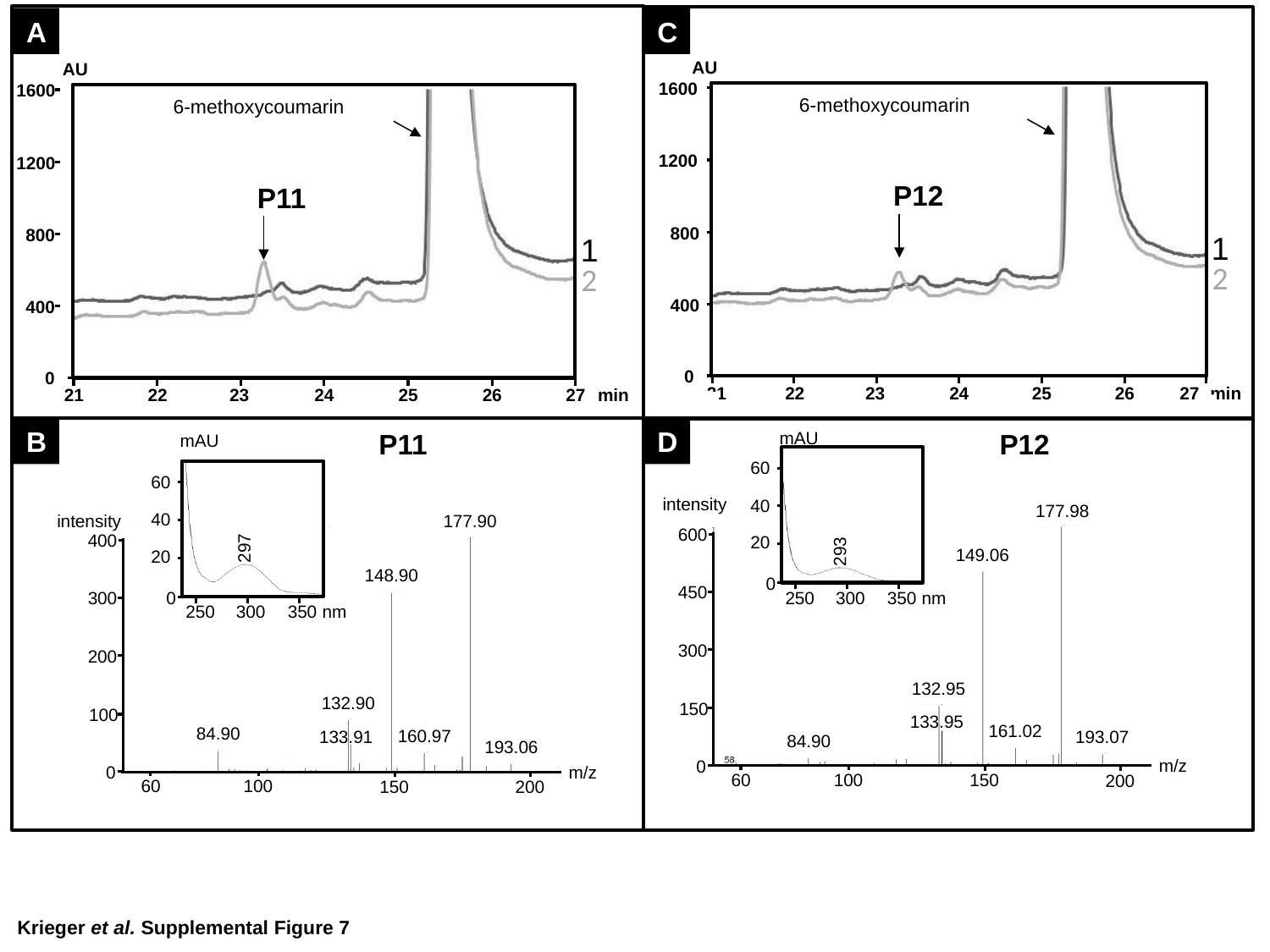

A
C
AU
AU
1600
1600
6-methoxycoumarin
P12
1
2
22
23
24
25
26
27
min
6-methoxycoumarin
1200
1200
P11
800
800
1
2
400
400
0
0
21
21
22
23
24
25
26
27
min
B
D
P11
P12
mAU
60
40
293
20
0
250
300
350
nm
mAU
60
40
297
20
0
250
300
350
nm
intensity
intensity
177.90
400
148.90
300
200
132.90
100
84.90
160.97
133.91
193.06
m/z
0
60
100
150
200
177.98
600
149.06
450
300
132.95
150
133.95
161.02
193.07
84.90
m/z
0
60
100
150
200
Krieger et al. Supplemental Figure 7

## Slide 14
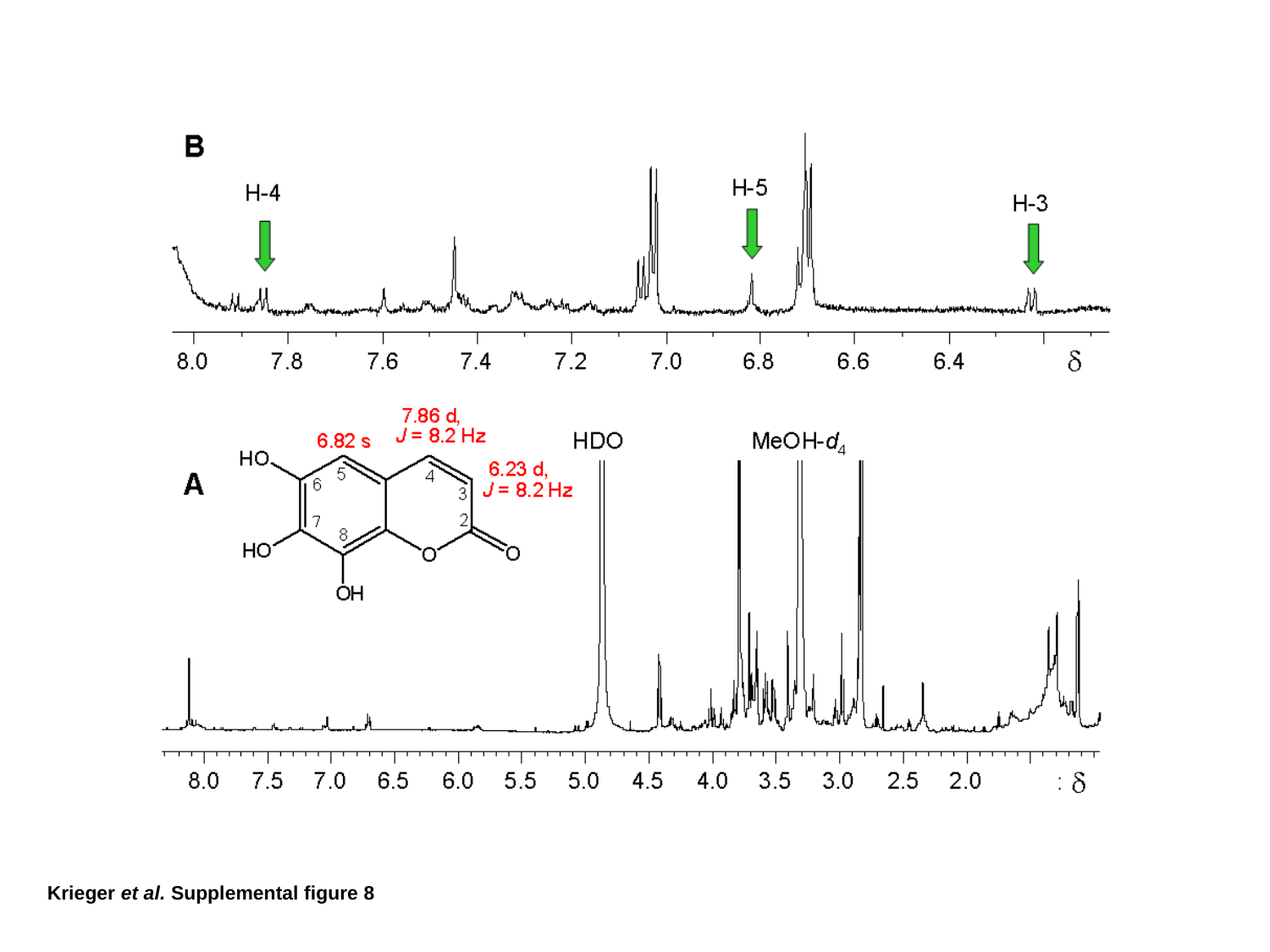

Krieger et al. Supplemental figure 8

## Slide 15
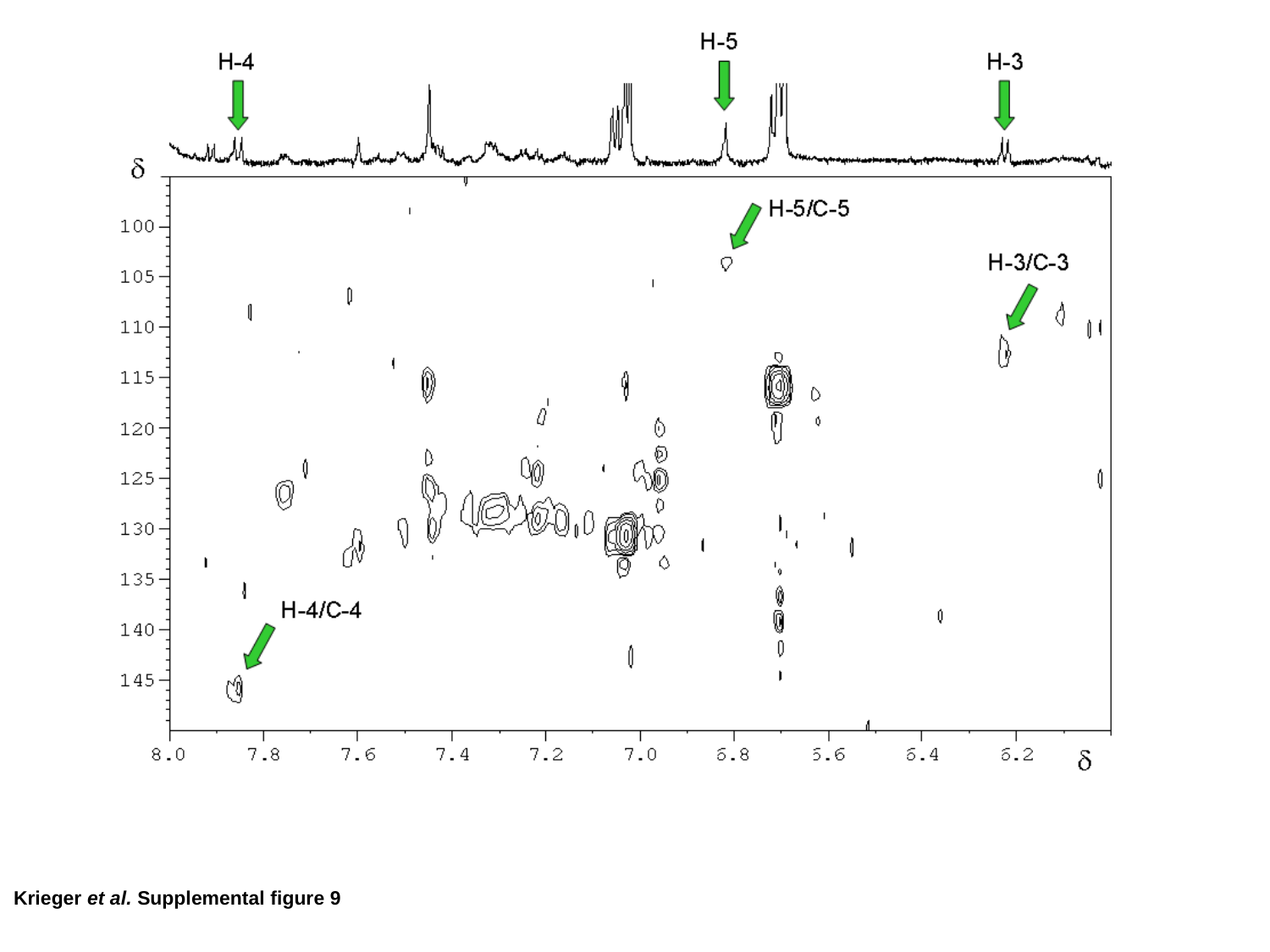

Krieger et al. Supplemental figure 9

## Slide 16
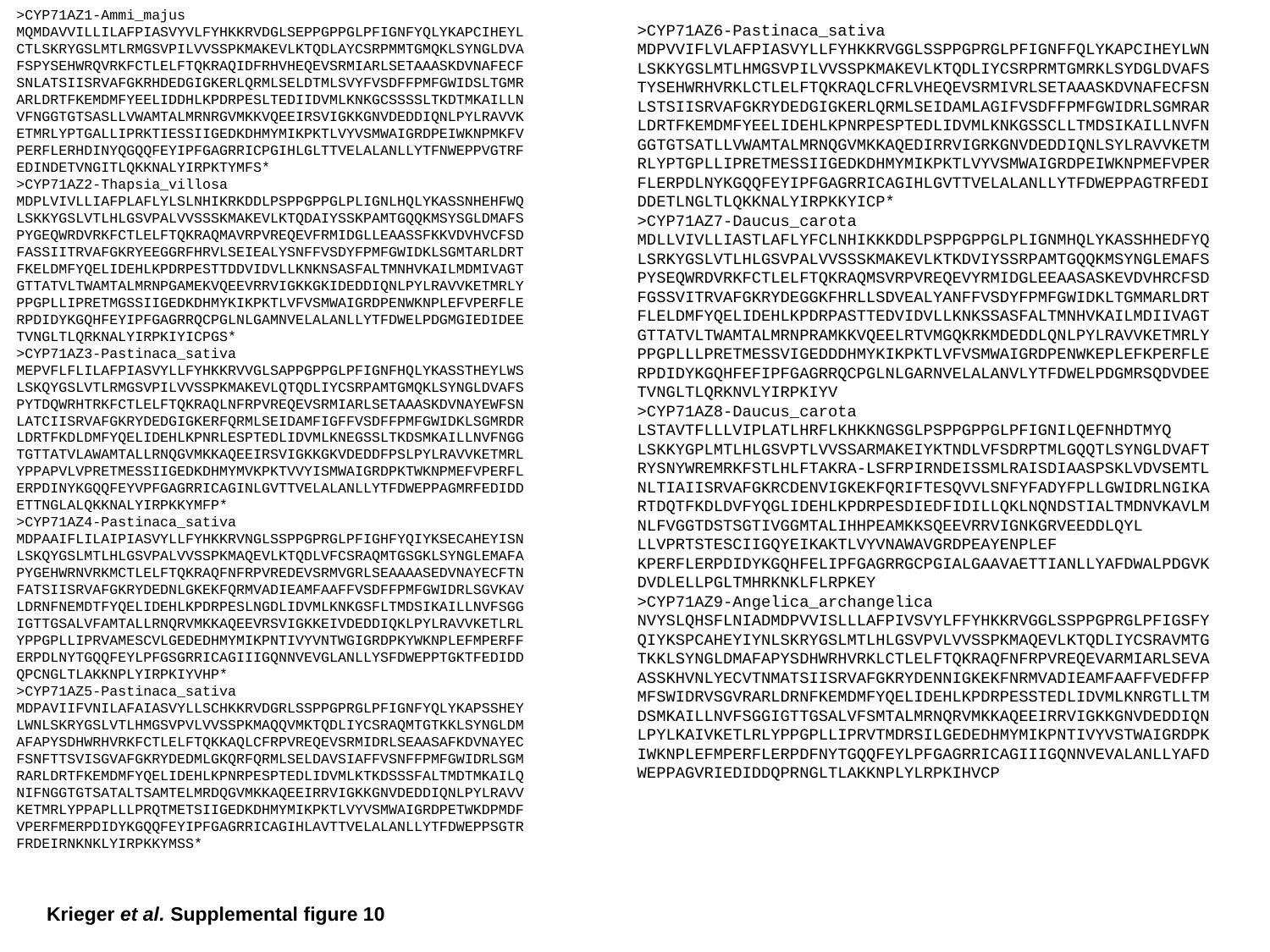

>CYP71AZ1-Ammi_majus
MQMDAVVILLILAFPIASVYVLFYHKKRVDGLSEPPGPPGLPFIGNFYQLYKAPCIHEYL
CTLSKRYGSLMTLRMGSVPILVVSSPKMAKEVLKTQDLAYCSRPMMTGMQKLSYNGLDVA
FSPYSEHWRQVRKFCTLELFTQKRAQIDFRHVHEQEVSRMIARLSETAAASKDVNAFECF
SNLATSIISRVAFGKRHDEDGIGKERLQRMLSELDTMLSVYFVSDFFPMFGWIDSLTGMR
ARLDRTFKEMDMFYEELIDDHLKPDRPESLTEDIIDVMLKNKGCSSSSLTKDTMKAILLN
VFNGGTGTSASLLVWAMTALMRNRGVMKKVQEEIRSVIGKKGNVDEDDIQNLPYLRAVVK
ETMRLYPTGALLIPRKTIESSIIGEDKDHMYMIKPKTLVYVSMWAIGRDPEIWKNPMKFV
PERFLERHDINYQGQQFEYIPFGAGRRICPGIHLGLTTVELALANLLYTFNWEPPVGTRF
EDINDETVNGITLQKKNALYIRPKTYMFS*
>CYP71AZ2-Thapsia_villosa
MDPLVIVLLIAFPLAFLYLSLNHIKRKDDLPSPPGPPGLPLIGNLHQLYKASSNHEHFWQ
LSKKYGSLVTLHLGSVPALVVSSSKMAKEVLKTQDAIYSSKPAMTGQQKMSYSGLDMAFS
PYGEQWRDVRKFCTLELFTQKRAQMAVRPVREQEVFRMIDGLLEAASSFKKVDVHVCFSD
FASSIITRVAFGKRYEEGGRFHRVLSEIEALYSNFFVSDYFPMFGWIDKLSGMTARLDRT
FKELDMFYQELIDEHLKPDRPESTTDDVIDVLLKNKNSASFALTMNHVKAILMDMIVAGT
GTTATVLTWAMTALMRNPGAMEKVQEEVRRVIGKKGKIDEDDIQNLPYLRAVVKETMRLY
PPGPLLIPRETMGSSIIGEDKDHMYKIKPKTLVFVSMWAIGRDPENWKNPLEFVPERFLE
RPDIDYKGQHFEYIPFGAGRRQCPGLNLGAMNVELALANLLYTFDWELPDGMGIEDIDEE
TVNGLTLQRKNALYIRPKIYICPGS*
>CYP71AZ3-Pastinaca_sativa
MEPVFLFLILAFPIASVYLLFYHKKRVVGLSAPPGPPGLPFIGNFHQLYKASSTHEYLWS
LSKQYGSLVTLRMGSVPILVVSSPKMAKEVLQTQDLIYCSRPAMTGMQKLSYNGLDVAFS
PYTDQWRHTRKFCTLELFTQKRAQLNFRPVREQEVSRMIARLSETAAASKDVNAYEWFSN
LATCIISRVAFGKRYDEDGIGKERFQRMLSEIDAMFIGFFVSDFFPMFGWIDKLSGMRDR
LDRTFKDLDMFYQELIDEHLKPNRLESPTEDLIDVMLKNEGSSLTKDSMKAILLNVFNGG
TGTTATVLAWAMTALLRNQGVMKKAQEEIRSVIGKKGKVDEDDFPSLPYLRAVVKETMRL
YPPAPVLVPRETMESSIIGEDKDHMYMVKPKTVVYISMWAIGRDPKTWKNPMEFVPERFL
ERPDINYKGQQFEYVPFGAGRRICAGINLGVTTVELALANLLYTFDWEPPAGMRFEDIDD
ETTNGLALQKKNALYIRPKKYMFP*
>CYP71AZ4-Pastinaca_sativa
MDPAAIFLILAIPIASVYLLFYHKKRVNGLSSPPGPRGLPFIGHFYQIYKSECAHEYISN
LSKQYGSLMTLHLGSVPALVVSSPKMAQEVLKTQDLVFCSRAQMTGSGKLSYNGLEMAFA
PYGEHWRNVRKMCTLELFTQKRAQFNFRPVREDEVSRMVGRLSEAAAASEDVNAYECFTN
FATSIISRVAFGKRYDEDNLGKEKFQRMVADIEAMFAAFFVSDFFPMFGWIDRLSGVKAV
LDRNFNEMDTFYQELIDEHLKPDRPESLNGDLIDVMLKNKGSFLTMDSIKAILLNVFSGG
IGTTGSALVFAMTALLRNQRVMKKAQEEVRSVIGKKEIVDEDDIQKLPYLRAVVKETLRL
YPPGPLLIPRVAMESCVLGEDEDHMYMIKPNTIVYVNTWGIGRDPKYWKNPLEFMPERFF
ERPDLNYTGQQFEYLPFGSGRRICAGIIIGQNNVEVGLANLLYSFDWEPPTGKTFEDIDD
QPCNGLTLAKKNPLYIRPKIYVHP*
>CYP71AZ5-Pastinaca_sativa
MDPAVIIFVNILAFAIASVYLLSCHKKRVDGRLSSPPGPRGLPFIGNFYQLYKAPSSHEY
LWNLSKRYGSLVTLHMGSVPVLVVSSPKMAQQVMKTQDLIYCSRAQMTGTKKLSYNGLDM
AFAPYSDHWRHVRKFCTLELFTQKKAQLCFRPVREQEVSRMIDRLSEAASAFKDVNAYEC
FSNFTTSVISGVAFGKRYDEDMLGKQRFQRMLSELDAVSIAFFVSNFFPMFGWIDRLSGM
RARLDRTFKEMDMFYQELIDEHLKPNRPESPTEDLIDVMLKTKDSSSFALTMDTMKAILQ
NIFNGGTGTSATALTSAMTELMRDQGVMKKAQEEIRRVIGKKGNVDEDDIQNLPYLRAVV
KETMRLYPPAPLLLPRQTMETSIIGEDKDHMYMIKPKTLVYVSMWAIGRDPETWKDPMDF
VPERFMERPDIDYKGQQFEYIPFGAGRRICAGIHLAVTTVELALANLLYTFDWEPPSGTR
FRDEIRNKNKLYIRPKKYMSS*
>CYP71AZ6-Pastinaca_sativa
MDPVVIFLVLAFPIASVYLLFYHKKRVGGLSSPPGPRGLPFIGNFFQLYKAPCIHEYLWN
LSKKYGSLMTLHMGSVPILVVSSPKMAKEVLKTQDLIYCSRPRMTGMRKLSYDGLDVAFS
TYSEHWRHVRKLCTLELFTQKRAQLCFRLVHEQEVSRMIVRLSETAAASKDVNAFECFSN
LSTSIISRVAFGKRYDEDGIGKERLQRMLSEIDAMLAGIFVSDFFPMFGWIDRLSGMRAR
LDRTFKEMDMFYEELIDEHLKPNRPESPTEDLIDVMLKNKGSSCLLTMDSIKAILLNVFN
GGTGTSATLLVWAMTALMRNQGVMKKAQEDIRRVIGRKGNVDEDDIQNLSYLRAVVKETM
RLYPTGPLLIPRETMESSIIGEDKDHMYMIKPKTLVYVSMWAIGRDPEIWKNPMEFVPER
FLERPDLNYKGQQFEYIPFGAGRRICAGIHLGVTTVELALANLLYTFDWEPPAGTRFEDI
DDETLNGLTLQKKNALYIRPKKYICP*
>CYP71AZ7-Daucus_carota
MDLLVIVLLIASTLAFLYFCLNHIKKKDDLPSPPGPPGLPLIGNMHQLYKASSHHEDFYQ
LSRKYGSLVTLHLGSVPALVVSSSKMAKEVLKTKDVIYSSRPAMTGQQKMSYNGLEMAFS
PYSEQWRDVRKFCTLELFTQKRAQMSVRPVREQEVYRMIDGLEEAASASKEVDVHRCFSD
FGSSVITRVAFGKRYDEGGKFHRLLSDVEALYANFFVSDYFPMFGWIDKLTGMMARLDRT
FLELDMFYQELIDEHLKPDRPASTTEDVIDVLLKNKSSASFALTMNHVKAILMDIIVAGT
GTTATVLTWAMTALMRNPRAMKKVQEELRTVMGQKRKMDEDDLQNLPYLRAVVKETMRLY
PPGPLLLPRETMESSVIGEDDDHMYKIKPKTLVFVSMWAIGRDPENWKEPLEFKPERFLE
RPDIDYKGQHFEFIPFGAGRRQCPGLNLGARNVELALANVLYTFDWELPDGMRSQDVDEE
TVNGLTLQRKNVLYIRPKIYV
>CYP71AZ8-Daucus_carota
LSTAVTFLLLVIPLATLHRFLKHKKNGSGLPSPPGPPGLPFIGNILQEFNHDTMYQ
LSKKYGPLMTLHLGSVPTLVVSSARMAKEIYKTNDLVFSDRPTMLGQQTLSYNGLDVAFT
RYSNYWREMRKFSTLHLFTAKRA-LSFRPIRNDEISSMLRAISDIAASPSKLVDVSEMTL
NLTIAIISRVAFGKRCDENVIGKEKFQRIFTESQVVLSNFYFADYFPLLGWIDRLNGIKA
RTDQTFKDLDVFYQGLIDEHLKPDRPESDIEDFIDILLQKLNQNDSTIALTMDNVKAVLM
NLFVGGTDSTSGTIVGGMTALIHHPEAMKKSQEEVRRVIGNKGRVEEDDLQYL
LLVPRTSTESCIIGQYEIKAKTLVYVNAWAVGRDPEAYENPLEF
KPERFLERPDIDYKGQHFELIPFGAGRRGCPGIALGAAVAETTIANLLYAFDWALPDGVK
DVDLELLPGLTMHRKNKLFLRPKEY
>CYP71AZ9-Angelica_archangelica
NVYSLQHSFLNIADMDPVVISLLLAFPIVSVYLFFYHKKRVGGLSSPPGPRGLPFIGSFY
QIYKSPCAHEYIYNLSKRYGSLMTLHLGSVPVLVVSSPKMAQEVLKTQDLIYCSRAVMTG
TKKLSYNGLDMAFAPYSDHWRHVRKLCTLELFTQKRAQFNFRPVREQEVARMIARLSEVA
ASSKHVNLYECVTNMATSIISRVAFGKRYDENNIGKEKFNRMVADIEAMFAAFFVEDFFP
MFSWIDRVSGVRARLDRNFKEMDMFYQELIDEHLKPDRPESSTEDLIDVMLKNRGTLLTM
DSMKAILLNVFSGGIGTTGSALVFSMTALMRNQRVMKKAQEEIRRVIGKKGNVDEDDIQN
LPYLKAIVKETLRLYPPGPLLIPRVTMDRSILGEDEDHMYMIKPNTIVYVSTWAIGRDPK
IWKNPLEFMPERFLERPDFNYTGQQFEYLPFGAGRRICAGIIIGQNNVEVALANLLYAFD
WEPPAGVRIEDIDDQPRNGLTLAKKNPLYLRPKIHVCP
Krieger et al. Supplemental figure 10

## Slide 17
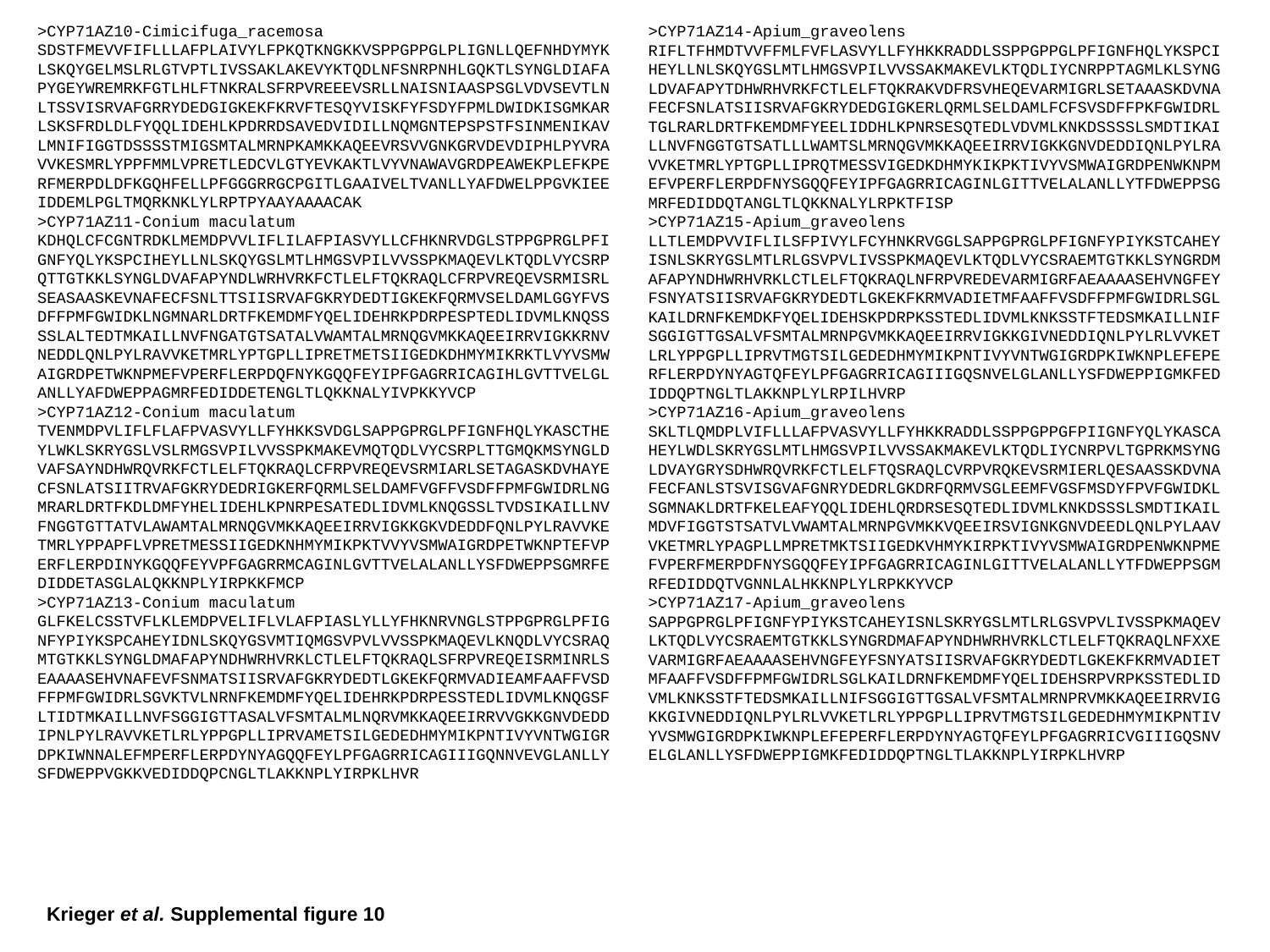

>CYP71AZ10-Cimicifuga_racemosa
SDSTFMEVVFIFLLLAFPLAIVYLFPKQTKNGKKVSPPGPPGLPLIGNLLQEFNHDYMYK
LSKQYGELMSLRLGTVPTLIVSSAKLAKEVYKTQDLNFSNRPNHLGQKTLSYNGLDIAFA
PYGEYWREMRKFGTLHLFTNKRALSFRPVREEEVSRLLNAISNIAASPSGLVDVSEVTLN
LTSSVISRVAFGRRYDEDGIGKEKFKRVFTESQYVISKFYFSDYFPMLDWIDKISGMKAR
LSKSFRDLDLFYQQLIDEHLKPDRRDSAVEDVIDILLNQMGNTEPSPSTFSINMENIKAV
LMNIFIGGTDSSSSTMIGSMTALMRNPKAMKKAQEEVRSVVGNKGRVDEVDIPHLPYVRA
VVKESMRLYPPFMMLVPRETLEDCVLGTYEVKAKTLVYVNAWAVGRDPEAWEKPLEFKPE
RFMERPDLDFKGQHFELLPFGGGRRGCPGITLGAAIVELTVANLLYAFDWELPPGVKIEE
IDDEMLPGLTMQRKNKLYLRPTPYAAYAAAACAK
>CYP71AZ11-Conium maculatum
KDHQLCFCGNTRDKLMEMDPVVLIFLILAFPIASVYLLCFHKNRVDGLSTPPGPRGLPFI
GNFYQLYKSPCIHEYLLNLSKQYGSLMTLHMGSVPILVVSSPKMAQEVLKTQDLVYCSRP
QTTGTKKLSYNGLDVAFAPYNDLWRHVRKFCTLELFTQKRAQLCFRPVREQEVSRMISRL
SEASAASKEVNAFECFSNLTTSIISRVAFGKRYDEDTIGKEKFQRMVSELDAMLGGYFVS
DFFPMFGWIDKLNGMNARLDRTFKEMDMFYQELIDEHRKPDRPESPTEDLIDVMLKNQSS
SSLALTEDTMKAILLNVFNGATGTSATALVWAMTALMRNQGVMKKAQEEIRRVIGKKRNV
NEDDLQNLPYLRAVVKETMRLYPTGPLLIPRETMETSIIGEDKDHMYMIKRKTLVYVSMW
AIGRDPETWKNPMEFVPERFLERPDQFNYKGQQFEYIPFGAGRRICAGIHLGVTTVELGL
ANLLYAFDWEPPAGMRFEDIDDETENGLTLQKKNALYIVPKKYVCP
>CYP71AZ12-Conium maculatum
TVENMDPVLIFLFLAFPVASVYLLFYHKKSVDGLSAPPGPRGLPFIGNFHQLYKASCTHE
YLWKLSKRYGSLVSLRMGSVPILVVSSPKMAKEVMQTQDLVYCSRPLTTGMQKMSYNGLD
VAFSAYNDHWRQVRKFCTLELFTQKRAQLCFRPVREQEVSRMIARLSETAGASKDVHAYE
CFSNLATSIITRVAFGKRYDEDRIGKERFQRMLSELDAMFVGFFVSDFFPMFGWIDRLNG
MRARLDRTFKDLDMFYHELIDEHLKPNRPESATEDLIDVMLKNQGSSLTVDSIKAILLNV
FNGGTGTTATVLAWAMTALMRNQGVMKKAQEEIRRVIGKKGKVDEDDFQNLPYLRAVVKE
TMRLYPPAPFLVPRETMESSIIGEDKNHMYMIKPKTVVYVSMWAIGRDPETWKNPTEFVP
ERFLERPDINYKGQQFEYVPFGAGRRMCAGINLGVTTVELALANLLYSFDWEPPSGMRFE
DIDDETASGLALQKKNPLYIRPKKFMCP
>CYP71AZ13-Conium maculatum
GLFKELCSSTVFLKLEMDPVELIFLVLAFPIASLYLLYFHKNRVNGLSTPPGPRGLPFIG
NFYPIYKSPCAHEYIDNLSKQYGSVMTIQMGSVPVLVVSSPKMAQEVLKNQDLVYCSRAQ
MTGTKKLSYNGLDMAFAPYNDHWRHVRKLCTLELFTQKRAQLSFRPVREQEISRMINRLS
EAAAASEHVNAFEVFSNMATSIISRVAFGKRYDEDTLGKEKFQRMVADIEAMFAAFFVSD
FFPMFGWIDRLSGVKTVLNRNFKEMDMFYQELIDEHRKPDRPESSTEDLIDVMLKNQGSF
LTIDTMKAILLNVFSGGIGTTASALVFSMTALMLNQRVMKKAQEEIRRVVGKKGNVDEDD
IPNLPYLRAVVKETLRLYPPGPLLIPRVAMETSILGEDEDHMYMIKPNTIVYVNTWGIGR
DPKIWNNALEFMPERFLERPDYNYAGQQFEYLPFGAGRRICAGIIIGQNNVEVGLANLLY
SFDWEPPVGKKVEDIDDQPCNGLTLAKKNPLYIRPKLHVR
>CYP71AZ14-Apium_graveolens
RIFLTFHMDTVVFFMLFVFLASVYLLFYHKKRADDLSSPPGPPGLPFIGNFHQLYKSPCI
HEYLLNLSKQYGSLMTLHMGSVPILVVSSAKMAKEVLKTQDLIYCNRPPTAGMLKLSYNG
LDVAFAPYTDHWRHVRKFCTLELFTQKRAKVDFRSVHEQEVARMIGRLSETAAASKDVNA
FECFSNLATSIISRVAFGKRYDEDGIGKERLQRMLSELDAMLFCFSVSDFFPKFGWIDRL
TGLRARLDRTFKEMDMFYEELIDDHLKPNRSESQTEDLVDVMLKNKDSSSSLSMDTIKAI
LLNVFNGGTGTSATLLLWAMTSLMRNQGVMKKAQEEIRRVIGKKGNVDEDDIQNLPYLRA
VVKETMRLYPTGPLLIPRQTMESSVIGEDKDHMYKIKPKTIVYVSMWAIGRDPENWKNPM
EFVPERFLERPDFNYSGQQFEYIPFGAGRRICAGINLGITTVELALANLLYTFDWEPPSG
MRFEDIDDQTANGLTLQKKNALYLRPKTFISP
>CYP71AZ15-Apium_graveolens
LLTLEMDPVVIFLILSFPIVYLFCYHNKRVGGLSAPPGPRGLPFIGNFYPIYKSTCAHEY
ISNLSKRYGSLMTLRLGSVPVLIVSSPKMAQEVLKTQDLVYCSRAEMTGTKKLSYNGRDM
AFAPYNDHWRHVRKLCTLELFTQKRAQLNFRPVREDEVARMIGRFAEAAAASEHVNGFEY
FSNYATSIISRVAFGKRYDEDTLGKEKFKRMVADIETMFAAFFVSDFFPMFGWIDRLSGL
KAILDRNFKEMDKFYQELIDEHSKPDRPKSSTEDLIDVMLKNKSSTFTEDSMKAILLNIF
SGGIGTTGSALVFSMTALMRNPGVMKKAQEEIRRVIGKKGIVNEDDIQNLPYLRLVVKET
LRLYPPGPLLIPRVTMGTSILGEDEDHMYMIKPNTIVYVNTWGIGRDPKIWKNPLEFEPE
RFLERPDYNYAGTQFEYLPFGAGRRICAGIIIGQSNVELGLANLLYSFDWEPPIGMKFED
IDDQPTNGLTLAKKNPLYLRPILHVRP
>CYP71AZ16-Apium_graveolens
SKLTLQMDPLVIFLLLAFPVASVYLLFYHKKRADDLSSPPGPPGFPIIGNFYQLYKASCA
HEYLWDLSKRYGSLMTLHMGSVPILVVSSAKMAKEVLKTQDLIYCNRPVLTGPRKMSYNG
LDVAYGRYSDHWRQVRKFCTLELFTQSRAQLCVRPVRQKEVSRMIERLQESAASSKDVNA
FECFANLSTSVISGVAFGNRYDEDRLGKDRFQRMVSGLEEMFVGSFMSDYFPVFGWIDKL
SGMNAKLDRTFKELEAFYQQLIDEHLQRDRSESQTEDLIDVMLKNKDSSSLSMDTIKAIL
MDVFIGGTSTSATVLVWAMTALMRNPGVMKKVQEEIRSVIGNKGNVDEEDLQNLPYLAAV
VKETMRLYPAGPLLMPRETMKTSIIGEDKVHMYKIRPKTIVYVSMWAIGRDPENWKNPME
FVPERFMERPDFNYSGQQFEYIPFGAGRRICAGINLGITTVELALANLLYTFDWEPPSGM
RFEDIDDQTVGNNLALHKKNPLYLRPKKYVCP
>CYP71AZ17-Apium_graveolens
SAPPGPRGLPFIGNFYPIYKSTCAHEYISNLSKRYGSLMTLRLGSVPVLIVSSPKMAQEV
LKTQDLVYCSRAEMTGTKKLSYNGRDMAFAPYNDHWRHVRKLCTLELFTQKRAQLNFXXE
VARMIGRFAEAAAASEHVNGFEYFSNYATSIISRVAFGKRYDEDTLGKEKFKRMVADIET
MFAAFFVSDFFPMFGWIDRLSGLKAILDRNFKEMDMFYQELIDEHSRPVRPKSSTEDLID
VMLKNKSSTFTEDSMKAILLNIFSGGIGTTGSALVFSMTALMRNPRVMKKAQEEIRRVIG
KKGIVNEDDIQNLPYLRLVVKETLRLYPPGPLLIPRVTMGTSILGEDEDHMYMIKPNTIV
YVSMWGIGRDPKIWKNPLEFEPERFLERPDYNYAGTQFEYLPFGAGRRICVGIIIGQSNV
ELGLANLLYSFDWEPPIGMKFEDIDDQPTNGLTLAKKNPLYIRPKLHVRP
Krieger et al. Supplemental figure 10

## Slide 18
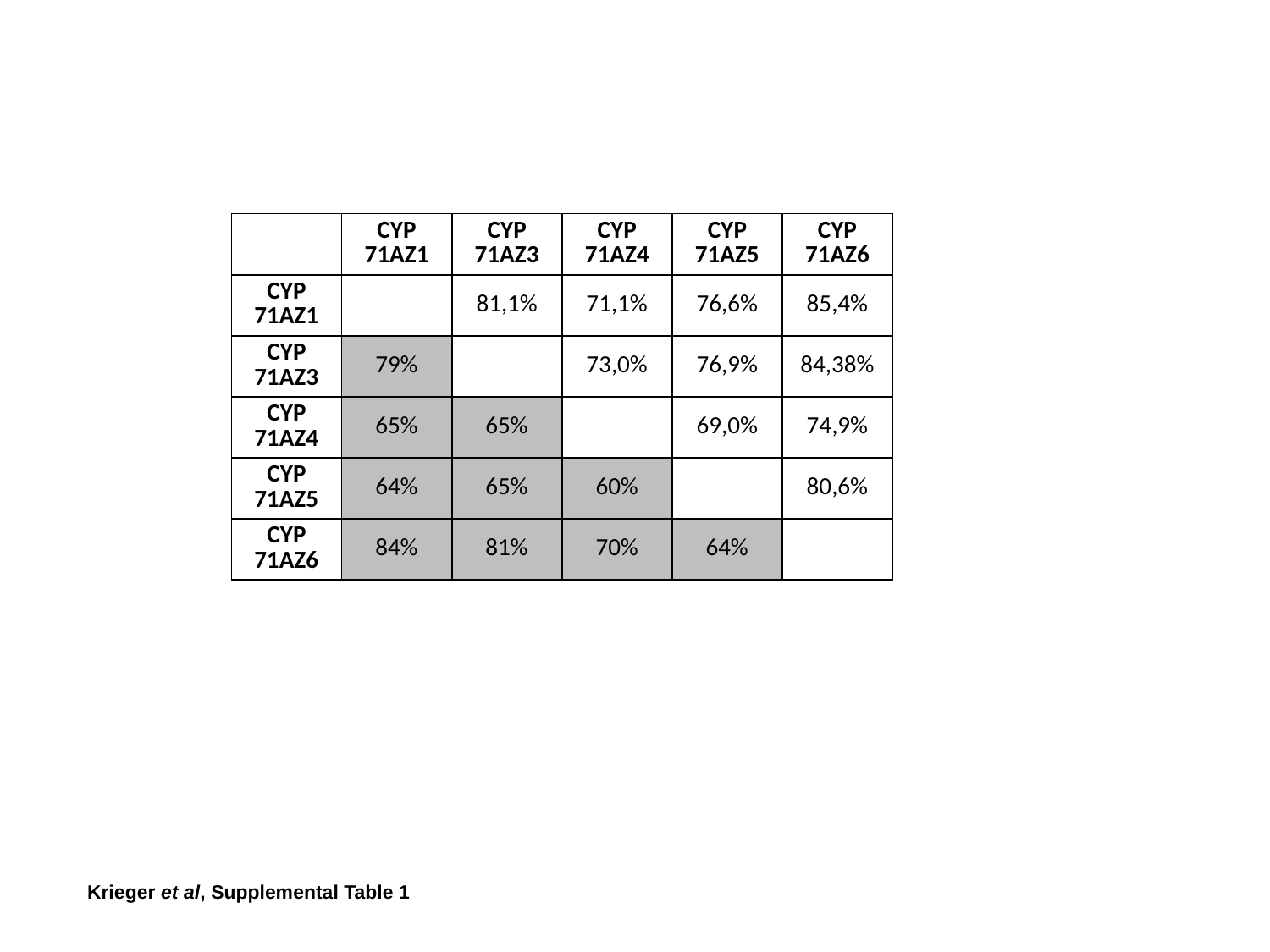

| | CYP 71AZ1 | CYP 71AZ3 | CYP 71AZ4 | CYP 71AZ5 | CYP 71AZ6 |
| --- | --- | --- | --- | --- | --- |
| CYP 71AZ1 | | 81,1% | 71,1% | 76,6% | 85,4% |
| CYP 71AZ3 | 79% | | 73,0% | 76,9% | 84,38% |
| CYP 71AZ4 | 65% | 65% | | 69,0% | 74,9% |
| CYP 71AZ5 | 64% | 65% | 60% | | 80,6% |
| CYP 71AZ6 | 84% | 81% | 70% | 64% | |
Krieger et al, Supplemental Table 1
